# Design of Functional Disorder in Charge-Transfer Cocrystals

**DOI:** 10.1021/acs.chemmater.5c02166

**Published:** 2025-10-27

**Authors:** Phoebe Eccles, Jesus Daniel Loya, Nina Aagaard, Abigail A. Moravek, Ren A. Wiscons

**Affiliations:** Department of Chemistry, 5798 Amherst College, 25 East Dr, Amherst, Massachusetts 01002, United States

## Abstract

In molecular crystals, disorder is often avoided or ignored as a defect; however, fundamental electronic phenomena, such as dielectricity and ferroelectricity, rely on motion present in the solid state for functionality. In these materials, crystallographic disorder can be an indicator of utility. Here, we explore the dynamics, electronic performance, and origin of whole-molecule disorder in a model charge-transfer (CT) cocrystal formed between 4,6-dimethyldibenzoselenophene (DMDBS) and 2,3-dichloro-5,6-dicyano-1,4-benzoquinone (DDQ), DMDBS-DDQ, to investigate the underlying energetics for design guidelines toward functional disorder in molecular crystals. DMDBS-DDQ was selected from the Cambridge Structural Database (CSD) for this investigation because DDQ is disordered over two positions related by a 180° rotation, coupling the disorder of DDQ to the inversion symmetry of the lattice. We prepare DMDBS-DDQ via single-crystal-to-single-crystal desolvation and demonstrate that the electrical performance and anisotropic thermal expansion behavior of the cocrystal are consistent with in-plane dynamic disorder above 33 °C (∼306 K). Importantly, we find that DMDBS-DDQ does not adhere to previous design principles regarding functional disorder in molecular systems that target size-mismatched molecular coformers to access high void/cavity space materials that may favor dynamic disorder. Instead, our findings suggest that design strategies toward dynamic disorder should be informed by interaction enthalpy surfaces of short-range intermolecular interactions.

## Graphical abstract

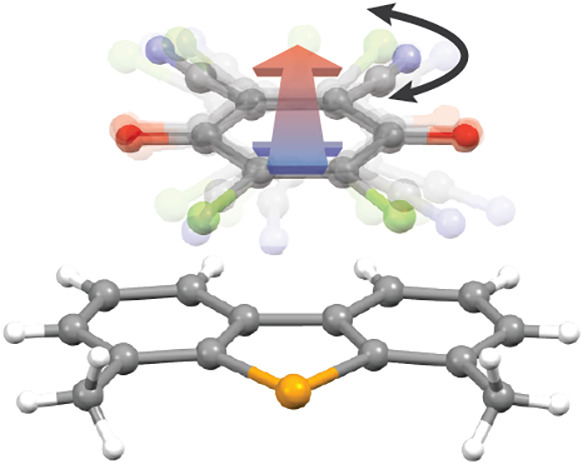

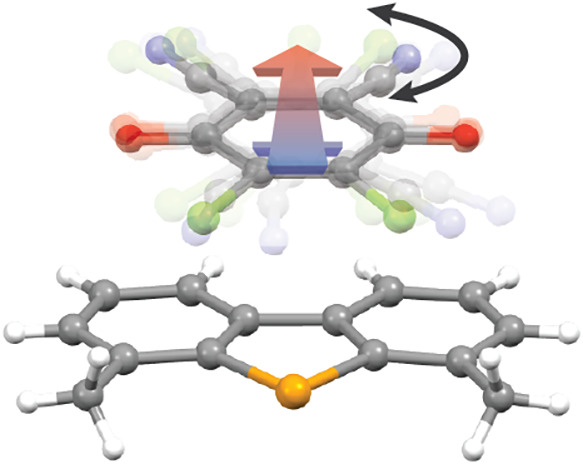

## 1 Introduction

Molecular crystals hosting whole-molecule rotational disorder have been targeted for applications as dielectrics in capacitors, sensors, transducers, and information storage materials.^1–11^ However, functional disorder (i.e., vibrations and/or rotations that can be driven by changes in temperature, pressure, and/or an applied electric field, *E*_field_) is challenging to intentionally introduce into crystal lattices as disorder is cited to arise from the presence of crystallographic void/cavity space or weak/nondirectional intermolecular interactions,^2,8,10,12,13^ factors that typically disfavor the formation of a stable crystal lattice. The incorporation of disorder into materials is further complicated by molecular and crystallographic symmetries, which have been shown to contribute to the energetics of dynamic disorder,^14,15^ a factor that remains difficult to predict *in silico* or to control in practice. Despite these challenges, guidelines to achieve disorder in molecular crystals have emerged from specific material case studies, such as combining multicomponent crystal coformers presenting a mismatch in molecular size to achieve crystals with high void space.^2,8,12^ Unfortunately, the generality of design guidelines toward functional disorder in molecular materials has not been demonstrated experimentally. Because the conditions under which whole-molecule disorder emerges in molecular crystals are closely related to factors that enthalpically destabilize the lattice, details of the disorder can offer insight into the interaction enthalpy landscape of the disordered species,^10,13^ which may inform the targeted design of functional disorder in molecular crystals.

This study investigates the dynamics, electronic performance, and origin of whole-molecule disorder in a model organic cocrystal formed between 4,6-dimethyldibenzoselenophene (DMDBS, Figure 1A) and 2,3-dichloro-5,6-dicyano-1,4-benzoquinone (DDQ, Figure 1A), DMDBS-DDQ. This system, first reported in 2019,^16^ was selected as a model system from the Cambridge Structural Database (CSD) because the single-crystal X-ray diffraction (SCXRD) structure features whole-molecule disorder of DDQ on a pseudosymmetrical lattice position. In the DMDBS-DDQ cocrystal, DDQ is disordered over two positions related by a 180° rotation (Figure 1B), suggesting that there are two favored orientations of DDQ separated by an energy barrier.^15^ This structural feature couples the DDQ disorder to the inversion symmetry of the lattice such that, theoretically, *E*_field_-driven in-plane rotation of DDQ can break the centrosymmetry of the crystal structure (Figure 1B).^1,2,4,8,17^ To achieve this functionality, the whole-molecule disorder in DMDBS-DDQ must arise from thermally- and/or electrically-accessible rotations and vibrations (dynamic disorder), which has not yet been characterized in this system.

**Figure 1:**
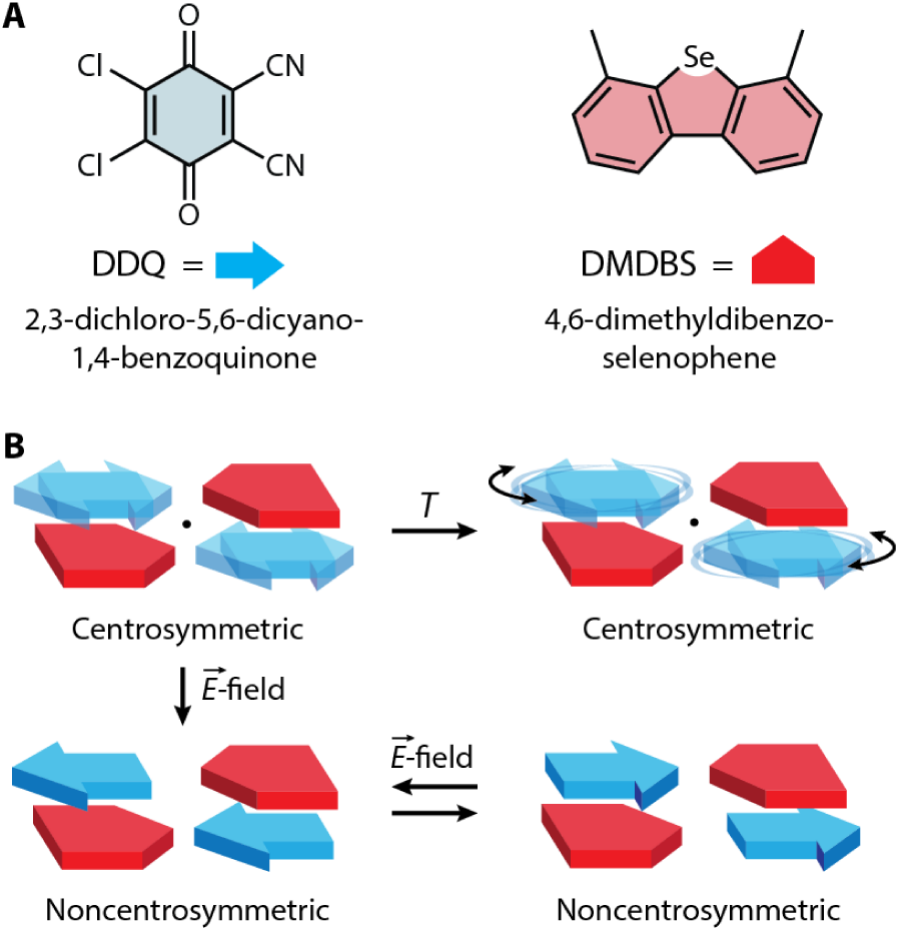
**A)** Skeletal representations of DDQ (left) and DMDBS (right) indicated by the blue arrow and red pentagon symbols in Figure 1B. **B)** (Top left) Diagrammatic representation of the DMDBS-DDQ crystal packing in which DDQ (blue) is disordered over two positions related by a 180° rotation, yielding an overall packing motif that is centrosymmetric (inversion center indicated by the black circle). (Top right) Upon heating the DMDBS-DDQ cocrystal, dynamic rotation of the DDQ molecules is predicted to take place, resulting in a centrosymmetric crystal packing motif. If DDQ molecules can be aligned with the application of an *E*_field_ (bottom left and right), the centrosymmetry of the crystal packing would be broken and the net polarization of the material could be inverted using a coercive *E*_field_.

In addition to the disorder presented by this system, the DMDBS-DDQ cocrystal was selected as a model system for the investigation of design principles toward functional disorder because it is a charge-transfer (CT) cocrystal, generally displaying a combination of strong directional CT interactions along the π-stacking axis and relatively weak electrostatic interactions perpendicular to the CT axis.^18^ While not a stringent structural requirement, dynamic disorder often emerges in crystalline systems when there exists a significant difference in interaction strength along different axes of the lattice such that the system can be heated to disrupt select weak interactions, while the overall thermal stability of the system is preserved by relatively strong interactions.

The presence of whole-molecule disorder has been observed in the structures of other CT cocrystals containing DDQ; however, the frequency of DDQ-containing CT cocrystals demonstrating whole-molecule disorder of DDQ, to our knowledge, has not been surveyed. Our search of the CSD returned 27 DDQ-containing neutral CT cocrystals (see the Supporting Information and Section 3, for relevant CSD refcodes). Of these structures, six demonstrate whole-molecule disorder of DDQ, yielding a frequency of DDQ disorder in neutral CT cocrystals of 22%. This frequency is challenging to contextualize with the crystallographic disorder frequency of other common CT acceptors (e.g., 2,3,5,6-tetrafluoro-7,7,8,8-tetracyanoquinodimethane, F_4_TCNQ) because CT acceptors do not typically adopt point group symmetries that allow for obvious crystallographically observable in-plane rotational disorder (e.g., DDQ is characterized by the *C*_2*v*_ point group, while F_4_TCNQ is *D*_2*h*_). However, DDQ is a member of the *C*_2*v*_-symmetric D*X*Q class of organic CT acceptors, which includes the unhalogenated (DCQ), dibrominated (DBQ), and diiodinated (DIQ) analogues of the 2,3-dicyano-1,4-benzoquinones and all three of these analogues show a crystallographic disorder frequency in neutral CT cocrystals of 0% (see Section 3). We also compared the DDQ crystallographic disorder frequency to that of tetrachlorophthalonitrile (TCPN), a small organic *C*_2*v*_-symmetric molecule that has been shown to dynamically rotate in cocrystals formed with this compound.^8^ A CSD search returned seven cocrystal structures containing TCPN, 5 (71%) of which show whole-molecule disorder of TCPN. Given that the frequency of TCPN disorder in cocrystals is likely biased 1) by the small sample size and 2) because four of the five disordered TCPN cocrystal structures are associated with one study exploring dynamic rotation of TCPN,^8^ the TCPN disorder frequency of a larger data set is likely lower than 71%. Collectively, this analysis demonstrates that, among the D*X*Q family of CT acceptors, DDQ shows a notable propensity toward forming cocrystals demonstrating whole-molecule disorder.

Herein, we prepare the DMDBS-DDQ cocrystal and demonstrate that the electrical performance and anisotropic thermal expansion behavior of the cocrystal is consistent with in-plane dynamic disorder above 33 °C (∼306 K). Upon analyzing the DMDBS-DDQ cocrystal structure and comparing the structure against those of the DMDB*Y*-D*X*Q (*Y* = chalcogen and *X* = halogen) family of cocrystals,^16,19^ we find that crystallographic void space is an imperfect predictor of whole-molecule rotational disorder as void space can indicate the presence of strong and directional interactions that can rotationally lock molecules, preventing functional dynamic disorder. It is critical to note that we differentiate between crystallographic void space (periodic unoccupied volume that arises due to the crystal packing) and nonperiodic immobile vacancies (defects), which have been correlated with orientational disordering (rotation).^20^ Instead, our findings suggest that design strategies toward dynamic disorder should be informed by interaction enthalpy surfaces of short-range intermolecular interactions. We find that, despite the low void volume in DMDBS-DDQ, this structure presents interaction distances that deviate significantly from idealized geometries, offering a parameter by which to rapidly screen the database of known disordered crystal structures for dynamic disorder, and introducing a high-throughput method by which to search for novel dielectric candidates.

## 2 Experimental Section

### 2.1 Materials

All reagents were used as received from suppliers without additional purification. DDQ (2,3-dichloro-5,6-dicyano-1,4-benzoquinone), *o*-iodotoluene, copper­(I) iodide (CuI), tribasic potassium phosphate (K_3_PO_4_), palladium­(II) trifluoroacetate (Pd^II^(tfa)_2_), pivalic acid (PivOH), Se^0^ powder, PEG_500_, silver­(I) acetate (AgOAc), sodium borohydride (NaBH_4_), sodium *tert*-butoxide (NaO^*t*^Bu), anhydrous magnesium sulfate (MgSO_4_), potassium carbonate (K_2_CO_3_), anhydrous methanol, and dichloromethane (DCM) were obtained from Sigma-Aldrich. Hexanes, tetrahydrofuran, and acetonitrile were obtained from Alfa Aesar. Acetonitrile used for crystallization was dried over 3 Å molecular sieves for at least 1 day prior to use.

### 2.2 Synthesis of Di-*o*-Tolyl Selane (1a) and Di-*o*-Tolyl Diselenide (1b)

Synthesis of 4,6-dimethyldibenzoselenophene (DMDBS) was adapted from a previously reported literature procedure.^16,19^ Se^0^ powder (781.3 mg, 9.893 mmol) was reacted with *o*-iodotoluene (4335 mg, 19.88 mmol) in PEG_500_ (16 mL) in the presence of CuI (209.4 mg, 1.099 mmol) and tribasic K_3_PO_4_ (9,960 mg, 46.92 mmol) in a sealed 100 mL pear-shaped flask. The flask was heated to 125 °C in a silicone oil bath and stirred for 24 h. The crude product was extracted with hexanes, washed with water, and then dried over anhydrous MgSO_4_. The material was concentrated to produce a yellow oil (mixture of **1a** and **1b**) and purified further using NaBH_4_ (*vide infra*). Selane intermediate **1a**: ^1^H NMR (400 MHz, acetone-*d*_6_) *δ*7.30 ppm (*d*, 2H), *δ*7.25 ppm (*t*, 2H), *δ*7.20 ppm (*d*, 2H), *δ*7.09 ppm (*t*, 2H), *δ*2.81 ppm (*s*, 6H). GCMS *o*-iodotoluene (retention time, rt 7.518 min, *m*/*z* 218), selane intermediate **1a** (rt 18.937 min, *m*/*z* 262), diselenide biproduct **1b** (rt 20.336, *m*/*z* 342). GCMS chromatogram and ^1^H NMR spectra are provided in the Figures SI9 and SI10, respectively.

### 2.3 Purification of Di-*O*o-Tolyl Selane (1a) from Di-*o*-Tolyl Diselenide (1b)

The crude yellow oil from the previous synthetic step was mixed in a 100 mL round-bottom flask with 2 mol equiv of NaBH_4_ and 1 mol equiv of NaO^*t*^Bu in anhydrous methanol (10 mL) and THF (10 mL) under nitrogen atmosphere. The reaction was heated to 60 °C and stirred for 20 min. The mixture was swiftly washed via liquid–liquid extraction with hexanes and a solution of sodium thiosulfate and sodium hydroxide. The hexanes layer was dried over anhydrous MgSO_4_ and concentrated under reduced pressure to yield a pink oil. The pink oil was purified by column chromatography on silica gel using a 10% (v/v) DCM in hexanes as the mobile phase. Column fractions were combined and concentrated to yield **1a** as a colorless oil.

### 2.4 Synthesis of 4,6-Dimethyldibenzoselenophene (DMDBS)

The selane intermediate **1a** (300.0 mg, 1.15 mmol) was reacted with K_2_CO_3_ (317.8 mg, 2.30 mmol), AgOAc (19.2 mg, 0.115 mmol), and Pd­(tfa)_2_ (192.3 mg, 0.575 mmol) in PivOH (2 mL) in a sealed 100 mL round-bottom flask under nitrogen atmosphere. The reaction was stirred for 16 h at 120 °C. The product was extracted into diethyl ether and filtered through a Celite pad. It was then washed with a solution of sodium bicarbonate and dried over anhydrous MgSO_4_. The crude product was purified by column chromatography on silica gel using a 10% (v/v) DCM in hexanes as the mobile phase. Column fractions were combined and concentrated to yield DMDBS. DMDBS: ^1^H NMR (400 MHz, acetone-*d*_6_) *δ*8.10 ppm (*d*, 2H), *δ*7.46 ppm (*t*, 2H), *δ*7.30 ppm (*d*, 2H), *δ*2.81 ppm (*s*, 6H). The NMR spectrum (see Figure SI11) of the product matches the previously reported NMR spectra from the literature^16^ and structure was further confirmed by single crystal X-ray diffraction (see Figure SI25).

### 2.5 Preparation of DMDBS_*X*_-DDQ_*Y*_ Cocrystals

A physical mixture of four DMDBS_*X*_-DDQ_*Y*_ cocrystal phases (DMDBS-DDQ, DMDBS-DDQ-ACN_*X*_, DMDBS_3_-DDQ_2_, and DMDBS_5_-DDQ_4_) is produced through evaporative crystallization. DDQ (1 mg, 0.0044 mmol) and DMDBS (1 mg, 0.0039 mmol) were added to a 1.5 mL polypropylene centrifuge tube with 1.0 mL of acetonitrile, and sonicated to ensure full dissolution. The acetonitrile was slowly evaporated over a two-day period, yielding a mixture of DMDBS_*X*_-DDQ_*Y*_ cocrystal phases in the centrifuge tube. Single crystals of DMDBS-DDQ-ACN_*X*_ were prepared by dissolving DDQ and DMDBS in acetonitrile. DDQ (1 mg, 0.0044 mmol) and DMDBS (1 mg, 0.0039 mmol) were added to a 1-dram vial with 0.5 mL of acetonitrile and sonicated to ensure full dissolution. The vial was placed in a freezer (−20 °C) for 1 h. Single crystals of DMDBS-DDQ-ACN_*X*_ precipitated and the solvent was decanted. Crystals of DMDBS-DDQ-ACN_*X*_ were heated to 85 °C under air or nitrogen atmosphere for 20 min to produce DMDBS-DDQ.

### 2.6 Solid-State Characterization

The structures of the materials were determined by a combination of single-crystal and powder X-ray diffraction. X-ray diffraction images were collected using a Rigaku XtaLAB Synergy-i X-ray diffractometer configured in a kappa goniometer geometry. The diffractometer is equipped with a variable-temperature device and a PhotonJet-S microfocus Cu source (λ = 1.54187 Å) operated at 50 kV and 1 mA. X-ray intensities were measured at room temperature with the Bantam detector placed 44.00 mm from the sample. The data were processed with CrysAlisPro version 41_64.117a (Rigaku Oxford Diffraction). Single-crystal X-ray structures were determined in OLEX2 ^21^ using SHELXT^22^ and refined using SHELXL.^23^ All ORTEP diagrams are given in Section 14. For each measurement of the lattice parameters, the data collection strategy was designed to achieve 60% completeness (for a full *P*-1 SCXRD collection), an I/σ of 10, and a redundancy of 3. All lattice constants are given in Section 13. The thermal stability and desolvation of the materials were characterized by a combination of differential scanning calorimetry (DSC, Section 9), thermogravimetric analysis (Section 10), and infrared (IR) spectroscopy (Section 11). DSC traces were measured on a Mettler Toledo Instrument DSC 3^+^ equipped with a Hubber TC100 cooling system under nitrogen atmosphere (50 mL/min). All DSC experiments were run in hermetic aluminum DSC pans with a heating rate of either 5 °C/min or 10 °C/min, covering a temperature range of 25 to 175 °C. DSC traces were analyzed using METTLER STARe default data viewer. TGA experiments were performed on a Mettler Toledo instrument TGA 2, equipped with a Hubber mini chiller 300 cooling system, under nitrogen atmosphere (20 mL/min) with an aluminum pan at a heating rate of 10 °C/min. TGA traces were analyzed using METTLER STARe default data viewer. FTIR spectra were obtained using a Bruker Tensor 37 instrument equipped with an MIRacle^TM^ single reflection attenuated total reflectance (ATR) accessory with a diamond/ZnSe window from PIKE Technologies. For each spectrum, 32 scans of the background and sample were collected. Background subtractions were performed using OPUS v6.5 Build 6, 5, 92 (Bruker Optik).

### 2.7 Electrical Property Characterization

Single-crystal devices of desolvated DMDBS-DDQ cocrystals were prepared by immobilizing crystals on a 1 cm × 1 cm glass slide with minimal vacuum grease. Guided by the crystal face indexation package available in CrysAlis Pro (Rigaku), silver contacts (Dupont Micromax 4922N) were placed on opposite (001) faces of the immobilized DMDBS-DDQ single crystals to make a two-point capacitance measurement. Polarization hysteresis loops were collected using a Precision Multiferroic II Test System (Radiant Technologies, Inc.) using Vision Software and a variable-temperature four-point probe station (Linkam Scientific). Polarization hysteresis loops were collected between 22 and 75 °C and all presented polarization hysteresis loops were collected between ± 25 V and at 2 Hz (see SI12). Device dimensions were used to convert the measured capacitance into net polarization and the drive voltage into electric field.

### 2.8 Computational Methods

Single point energy calculations at ground state and in the gas phase were performed using Spartan’20.^24^ Atom positions were obtained from single-crystal X-ray diffraction data. DFT was used with the ωB97X-D exchange-correlation functional and the split valence basis set 6–31G­(*). Electrostatic potential maps were computed and mapped onto an isodensity surface at 0.002 e/au^3^ with medium resolution. Crystallographic void space was measured to quantify the packing density/coefficient of the cocrystals. This was performed using the visualization and analysis software Mercury^25,26^ from the Cambridge Crystallographic Data Centre (CCDC). The Pore Analyzer tool was used with a probe radius of 0.53 Å and grid spacing of 0.3 Å to obtain the void space of the cocrystal structures.

## 3 Results and Discussion

### 3.1 The DMDBS-DDQ Cocrystal Structure

The DMDBS-DDQ cocrystal was first reported in 2019 among a family of related neutral CT crystals, DMDB*Y*-D*X*Q,^16,19^ of which DMDBS-DDQ is the only member that is not crystallographically isostructural (Figure 2A). In general, the degree of charge-transfer (*ρ*) in CT cocrystals varies in strength from neutral (0 electrons, e, < *ρ* ≤ 0.5 e) to ionic (0.5 e < *ρ* ≤ 1.0 e).^16,18^ The higher proportion of neutral CT cocrystals that display characteristics of dynamic disorder (relative to ionic CT cocrystals) has been rationalized by the CT interaction strength in neutral CT systems being less sensitive to the extent of overlap between the orbitals participating in the CT interaction, leading neutral CT cocrystals to be more “tolerant” of in-plane dynamics.^2,10^ For this reason, the CT neutrality of the DMDB*Y*-D*X*Q family of cocrystals is taken to be an advantage toward the investigation of disorder dynamics in DMDBS-DDQ.

**Figure 2:**
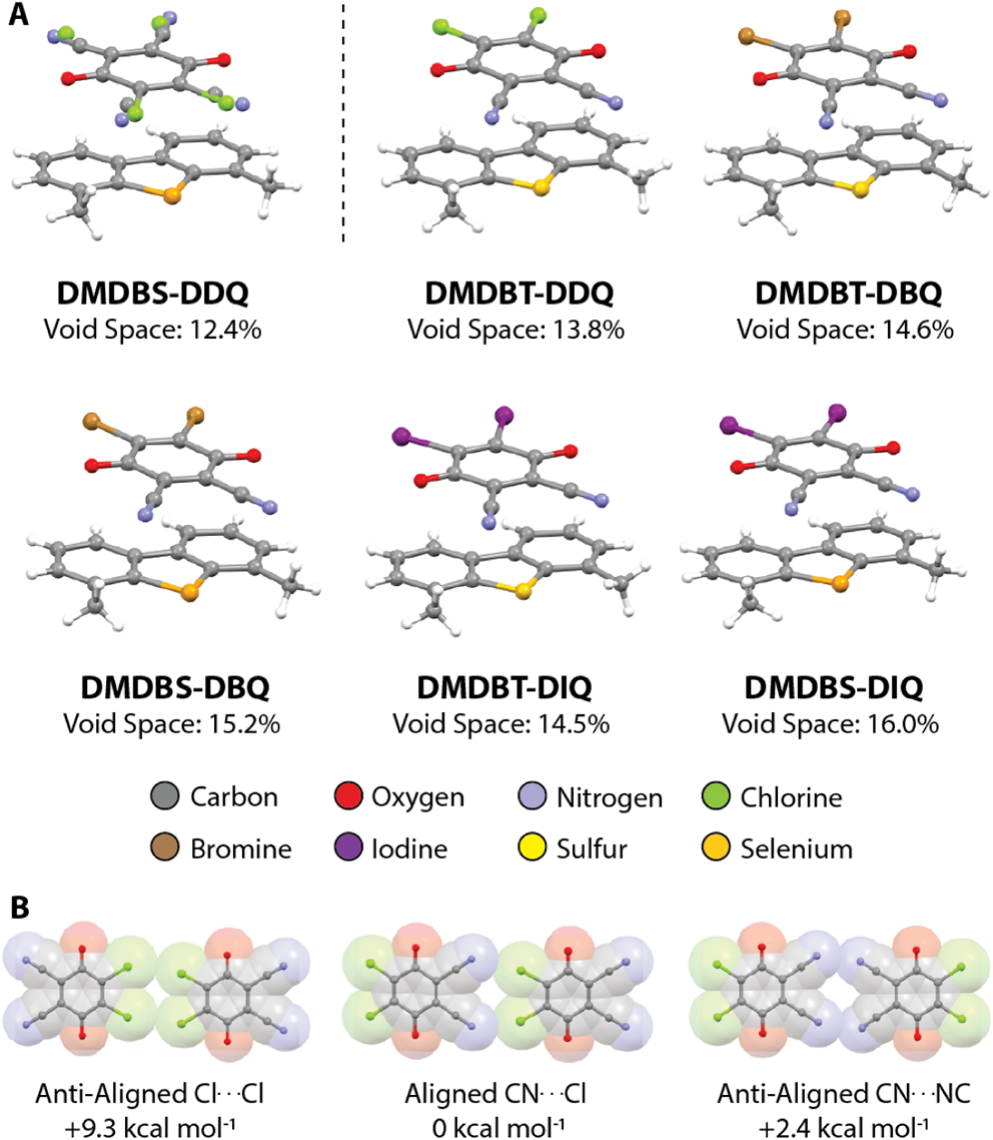
**A)** CT interaction geometries in the DMDB*Y*-D*X*Q family of cocrystals taken from the CSD. The top-left structure is DMDBS-DDQ (FOXCAZ, *P*-1). The remaining structures all solve in the *Cmc*2_1_ space group: DMDBT-DDQ (FOXDAA), DMDBT-DBQ (FOXCUT), DMDBS-DBQ (FOXBUS), DMDBT-DIQ (KUQWIF), DMDBS-DIQ (KUQZAA). DMDBT = 4,6-dimethyldibenzothiophene, DBQ = 2,3-dibromo-5,6-dicyano-1,4-benzoquinone, DIQ = 2,3-diiodo-5,6-dicyano-1,4-benzoquinone. **B)** In-plane interaction geometries and enthalpies (ωB97X-D/6–31G­(*)) for the three interaction motifs made possible by the 180° rotational disorder for DDQ: antialigned Cl···Cl interaction (left), aligned CN···Cl interaction (middle), and antialigned CN···NC interaction (right).

The dominant structure type presented by DMDB*Y*-D*X*Q cocrystals (with the exception of DMDBS-DDQ) is characterized by alternation of the DMDB*Y* and D*X*Q coformers along the π-stacking direction and interactions between π-stacking columns that are reinforced by halogen bonding interactions (-*Y*···*X* and -C≡N···*X*). These in-plane halogen bonding interactions align the D*X*Q and DMDB*Y* molecules such that the overall packing motif is noncentrosymmetric, solving in the *Cmc*2_1_ space group. Similarly to the dominant DMDB*Y*-D*X*Q structure type, DMDBS-DDQ also adopts an alternating π-stacking motif between DMDBS and DDQ; however, the halogen bonding interaction geometries differ significantly. Because DDQ molecules are disordered over two positions related by a 180° rotation, three distinct in-plane interaction geometries are allowed (Figure 2B). Determination of the interaction enthalpies (ωB97X-D/6–31G (*)) for the three in-plane interaction geometries allowed by the 180° DDQ rotational disorder suggests a preference for parallel alignment of in-plane DDQ homodimers (Figure 2B). However, modeling of the whole-molecule disorder leads the structure to solve as crystallographically centrosymmetric, refining in *P*-1. Given the coupling between the crystallographic centrosymmetry and the DDQ whole-molecule rotational disorder, we prepared the DMDBS-DDQ cocrystal to investigate the possibility of electronically polarizing the material and interconverting the net polarization of the material with an electric field.

### 3.2 DMDBS-DDQ Cocrystal Formation by SCSC Desolvation

DMDBS-DDQ is reportedly crystallized from acetonitrile by evaporation;^16^ however, we find that this method yields a physical mixture of multiple crystal forms, three of which were unreported. The crystal structures of these unreported solid forms were determined by SCXRD. In addition to the target 1:1 DMDBS-DDQ cocrystal, an isostructural DMDBS-DDQ cocrystal solvated with channels of disordered acetonitrile molecules (DMDBS-DDQ-ACN_*X*_, where 0 ≤ *X* < 1) crystallizes, as well as a cocrystals with a 3:2 molar ratio of DMDBS and DDQ, respectively, DMDBS_3_-DDQ_2_, and a 5:4 molar ratio of DMDBS and DDQ, DMDBS_5_-DDQ_4_ (see SI14 for ORTEP diagrams). In all three unreported cocrystals, alternating CT stacks of DMDBS and DDQ and significant whole molecule disorder are present: disordered acetonitrile and DDQ in DMDBS-DDQ-ACN_*X*_ and disordered DMDBS in DMDBS_3_-DDQ_2_ and DMDBS_5_-DDQ_4_ (DDQ is not disordered in these two cocrystals). Additional discussion of DMDBS_3_-DDQ_2_ and DMDBS_5_-DDQ_4_ crystal packing is provided in SI5. The formation of these three undesired and disordered crystal forms suggests that interactions between DMDBS-DDQ units are relatively weak and motivated the development of an alternative crystallization approach.

DMDBS-DDQ-ACN_*X*_ selectively crystallizes from saturated acetonitrile solution at low temperature (−20 °C). Given the similarity in crystal packing motif between DMDBS-DDQ-ACN_*X*_ and the target DMDBS-DDQ cocrystal, the possibility of accessing phase-pure DMDBS-DDQ in bulk via single-crystal-to-single-crystal (SCSC) desolvation of DMDBS-DDQ-ACN_*X*_ was explored by thermogravimetric analysis (TGA, see Figure 3A). The TGA trace reveals a mass loss event with an onset temperature of ∼ 70 °C, consistent with the loss of acetonitrile. This is supported by loss of the acetonitrile −CN vibrational mode in IR spectroscopy following heating (Figure 3B). The plateau in the TGA trace following the mass loss event indicates that ∼ 3% of the sample mass is lost as acetonitrile vapor, which is consistent with an initial occupancy of ∼37 mol %.

**Figure 3:**
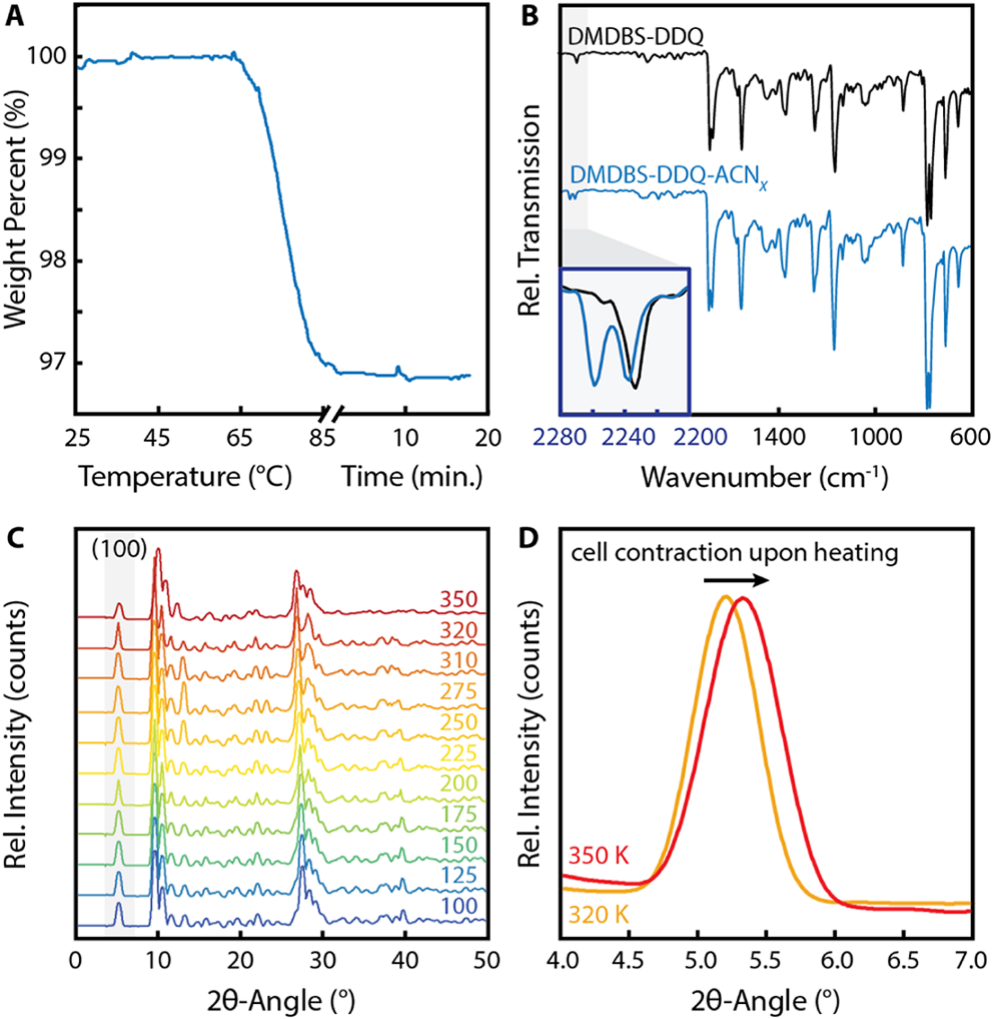
**A)** Thermogravimetric analysis (TGA) trace collected on DMDBS-DDQ-ACN_*X*_ showing a mass loss event ∼ 70 °C; **B)** infrared (IR) spectra collected on DMDBS-DDQ-ACN_*X*_ before (blue) and after (black) desolvation; the inset shows the nitrile region of both IR spectra; **C)** VT-PXRD patterns collected for DMDBS-DDQ-ACN_*X*_ at the temperatures in units of Kelvin indicated along the right axis; and **D)** the (100) diffraction peak of DMDBS-DDQ-ACN_*X*_ collected at 320 and 350 K, highlighting the cell contraction that takes place upon desolvation.

Structural determination of single crystals selected from the DMDBS-DDQ-ACN_*X*_ bulk sample before and after heating confirms SCSC desolvation to produce the desired DMDBS-DDQ cocrystal. Desolvation was also investigated for bulk DMDBS-DDQ-ACN_*X*_ using variable-temperature powder XRD (VT-PXRD). PXRD patterns (see Figure 3C) were collected at intervals between 100 (−173 °C) and 350 K (77 °C), revealing the expected shift in peak positions (see Figures 3D and Section 8) without significant increase in the FWHM of the peaks. The absence of significant peak broadening suggests preservation of the sample crystallinity and uniformity of desolvation across the bulk sample.

### 3.3 Electrical Characterization

Equipped with a reliable method to produce DMDBS-DDQ single crystals, the effect of the DDQ disorder on the electrical properties of DMDBS-DDQ was investigated. *E*_field_-driven polarization hysteresis loops (*P-E*) were measured between 295 – 348 K (22 – 75 °C) on single-crystalline devices of DMDBS-DDQ. Guided by the crystal face indexation package available in CrysAlis Pro (Rigaku), silver contacts were placed on the (001) faces of DMDBS-DDQ single crystals to measure the bulk electrical polarization across the *c*-axis of the material, approximately parallel to the in-plane DDQ disorder (see Figure 4). No polarization switching or saturation behavior was observed across the measured temperature range, indicating that DMDBS-DDQ is not a room-temperature ferroelectric;^17,27^ however, DMDBS-DDQ does demonstrate net polarization features of a lossy dielectric at room temperature that were further analyzed to investigate the electrical effect of the DDQ disorder.

**Figure 4:**
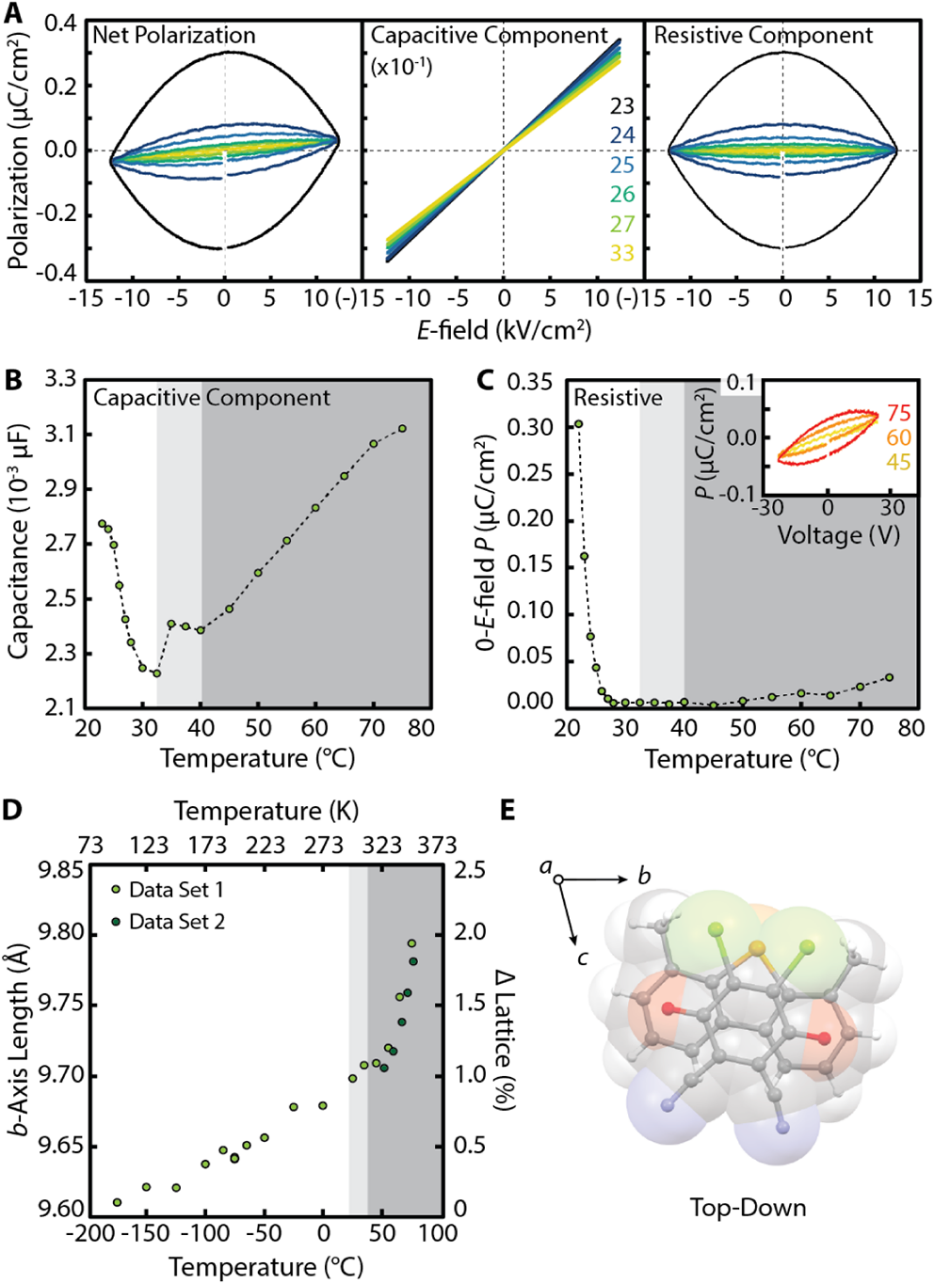
**A)** Net polarization hysteresis loops (left), the capacitive component of the polarization hysteresis loops (middle), and the resistive component of the polarization hysteresis loops measured at the temperatures in units of degrees Celsius indicated along the right axis of the center plot. **B)** Temperature-dependence of the capacitive component of the polarization hysteresis loops with the three discussed phases indicated in shades of gray. **C)** Temperature-dependence of the resistive component of the polarization hysteresis loops with an inset showing the reopening of the loop between 45 and 75 °C. **D)** *b*-axis length determined by VT-SCXRD between 100 – 350 K (−173 – 77 °C). **E)** Top-down view of a CT stacked DMDBS-DDQ dimer relative to the unit cell axes.

The capacitive (*C* ∝ d*P*/d*E*)^28^ and resistive (*R* ∝ *P*_0‑Field_ for a non-ferroelectric dielectric) components of the net “polarization” loops were separated and plotted as a function of temperature (see Figure 4A–C). Initially, the device demonstrates a decrease in the capacitance upon heating from 295 – 306 K (22 – 33 °C); this behavior can be rationalized by the thermal expansion term dominating the change in capacitance upon heating. Between 306 – 313 K (33 – 40 °C), there is a rapid increase and plateau in the capacitance. In the absence of thermal contraction, the discontinuity in the capacitance suggests accessibility of vibrational modes that increase the *c*-axis dielectric constant (e.g., in-plane rotation and alignment of DDQ),^29,30^ while the plateau in the thermal dependence of the capacitance indicates that neither the unit cell parameters nor the dielectric constant vary significantly between 35 and 40 °C. This plateau can be understood as an intermediate structural phase in which the in-plane rotation is thermally locked but interconversion between the two disordered states can be driven by the *E*_field_. At temperatures above 313 K (40 °C), the capacitance steadily increases with temperature, consistent with an increase in the *c*-axis dielectric constant due to freeing of the in-plane rotation of DDQ.

The resistive component of the net polarization loop, proportional to the zero *E*_field_ polarization magnitude (*P*_0‑Field_), also demonstrates temperature dependence that can be characterized by the three phases: between 295 – 306 K, 306 – 313 K, and above 313 K. While the *P*_0‑Field_ appears to initially decrease and plateau before increasing upon heating, the collapse of the polarization hysteresis loop upon heating from room temperature can also result from a shift in the frequency regime of the resistive component with changing temperature. This complex behavior is the result of changes in both the molecular and electronic structures of the material and requires further investigation to decisively connect the resistance changes to possible in-plane rotational behavior of DDQ.

### 3.4 Thermal Expansion Anisotropy

Because interpretation of the capacitive component of the polarization hysteresis loops strongly depends on the thermal expansion behavior of the material, variable-temperature SCXRD data were measured for DMDBS-DDQ single crystals between 100 – 378 K (−173 – 95 °C). In addition, thermal expansion anisotropy can assist in separating structural features that contribute to dynamic and static disorder and has been analyzed to corroborate molecular mechanisms of dielectricity.^7,9,31^ We observe that, under the conditions used to control the SCXRD sample collection temperature, rapid sublimation of the DMDBS-DDQ cocrystal is promoted at temperatures above 350 K (77 °C). This is consistent with a subtle endotherm in the differential scanning calorimetry (DSC) trace of the desolvated DMDBS-DDQ cocrystal that is revealed as a transition with an onset at 80 °C (upon heating) in the first-derivative of the DSC trace (see Section 9). For data collected between 100 – 350 K, the unit cell parameters were refined and plotted as a function of the data collection temperature.

We exclusively observe positive thermal expansion along all three crystallographic axes across the measured temperature range (see SI13), supporting our interpretation of the capacitive component of the measured polarization hysteresis loops. Notably, we measure significant change in the *b*-axis thermal expansion coefficient at 330 K (∼57 °C, see Figure 4D) in the absence of a significant change in the volumetric thermal expansion coefficient (see Section 13). This behavior is reproduced in multiple DMDBS-DDQ samples. Analysis of the DMDBS-DDQ SCXRD structure reveals that the *b*-axis approximately aligns with the narrow axis of the CT π-stacks when viewed down the CT axis (Figure 4E, “top-down” view). The agreement of temperature-dependent features identified in the electrical and structural measurements strongly support *E*_field_- and thermally-driven dynamic rotation of DDQ at elevated temperatures.

### 3.5 Insight into Disorder from Interaction Enthalpy Surfaces

Dielectric behavior via dynamic rotation is cited to arise from crystallographic void space in the CT cocrystals imparted through the size-mismatch of the CT coformers.^2,8^ To investigate whether size mismatch in CT coformers accounts for the differences in the solid-state packing and disorder of the DMDBS-DDQ cocrystal within the family of DMDB*Y*-D*X*Q cocrystals, void space was calculated for each DMDB*Y*-D*X*Q cocrystal using the Pore Analyzer tool available through Mercury (CCDC).^25,26^ The mismatch in coformer size was quantified as the ratio of volumes enclosed by an isosurface calculated for the crystallographic geometries of the separated CT coformers using Spartan’20 (Wavefunction).^24^ We find that periodic trends do successfully predict the relative volumes between the DMDB*Y* or D*X*Q analogues such that, expectedly, DMDBS-DDQ demonstrates the greatest mismatch in size between the CT coformers among all DMDB*Y* and D*X*Q combinations (see SI5). However, counter to published observations about dynamic disorder in molecular cocrystals, we find that DMDBS-DDQ demonstrates the lowest crystallographic void volume among all 1:1 DMDB*Y*-D*X*Q cocrystals (see Figures 5A and SI5). Furthermore, crystallographic void space was calculated for the crystal structures of DMDBS_3_-DDQ_2_ and DMDBS_5_-DDQ_4_, suggesting that decreasing DDQ content decreases void space and increases packing efficiency (see Section 4). We propose that crystallographic void space is an imperfect predictor of whole-molecule rotational disorder as void space can indicate the presence of strong and directional enthalpically favorable interactions that can rotationally lock molecules, preventing functional dynamic disorder.

**Figure 5:**
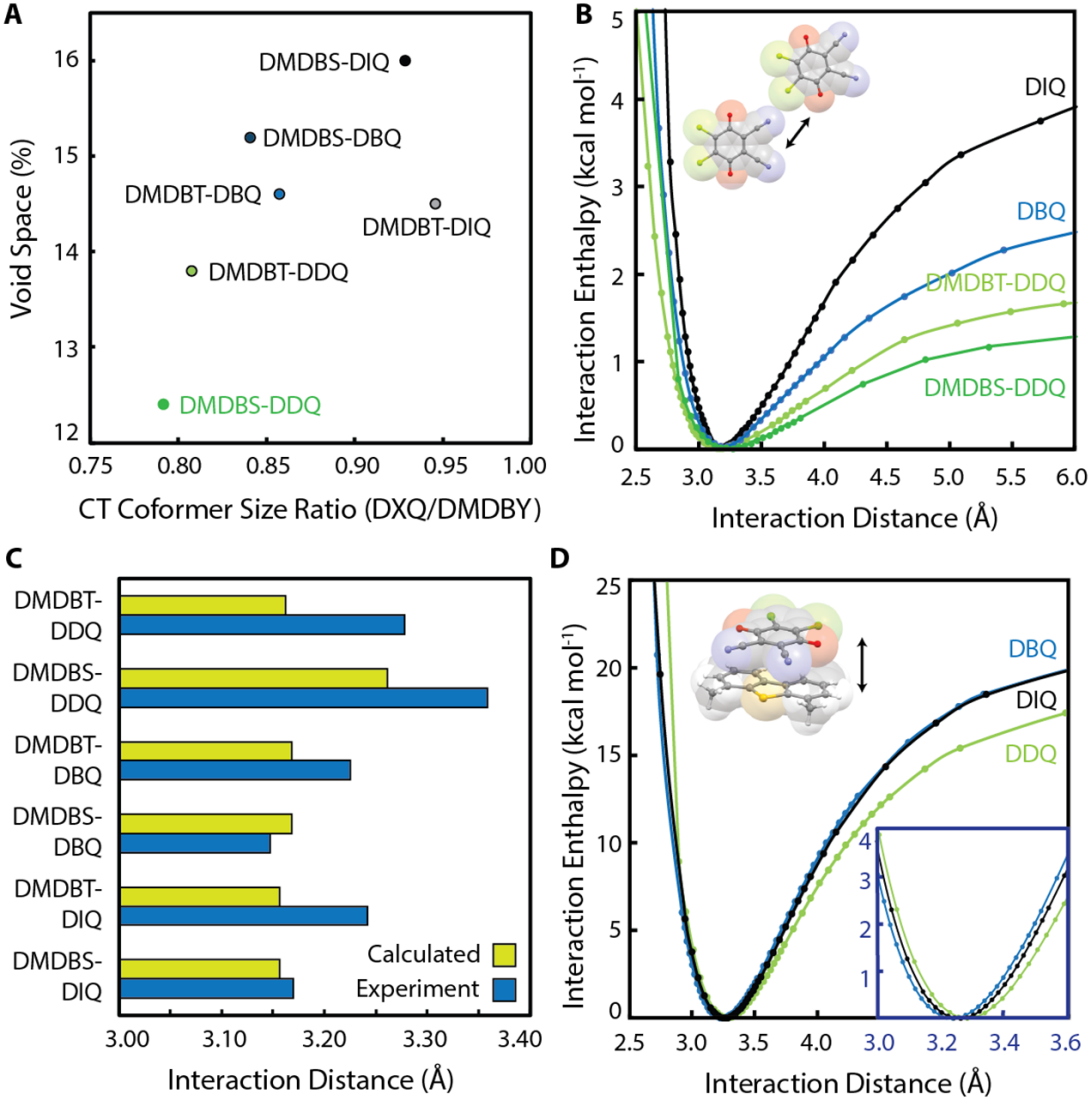
**A)** Plot of the CT coformer size ratio quantifying the size mismatch against the measured crystallographic void space for DMDB*Y*-D*X*Q cocrystals. Previous disorder guidelines suggest that a negative correlation should be present; however, this is not observed. **B)** Interaction enthalpy surfaces calculated for D*X*Q halogen-bonded homodimers for crystallographic geometries represented by the inset except for the DDQ homodimer calculated for DMDBS-DDQ; the halogen bonding interaction geometry used for DMDBS-DDQ is given in Figure 2B (center). **C)** Graph showing the calculated equilibrium (yellow) and experimental (blue) halogen bonding homodimer interaction distances for each DMDB*Y*-D*X*Q cocrystal. **D)** Interaction enthalpy surfaces calculated for DMDBS-D*X*Q CT heterodimers and the π-stacked CT interaction geometry (shown in inset).

The origin of crystallographic disorder in DMDBS-DDQ was further investigated by calculating interaction enthalpy surfaces (ωB97X-D/6–31G­(*)) for the interactions in the DMDB*Y*-D*X*Q cocrystals that contribute to the enthalpic barrier to in-plane rotation: halogen-bonded D*X*Q (X = Cl, Br, and I) homodimers (Figures 5B and SI4). We find that the interaction enthalpy surface is shallower for homodimers of DDQ and is steepest for DIQ (X = iodine) homodimers, indicative of the increasing halogen bonding strength with increasing halogen atom polarizability. Compared across all experimental DMDB*Y*-D*X*Q structures, the DMDBT-DDQ and DMDBS-DDQ homodimer halogen bonds are significantly longer, on average, than the calculated equilibrium interaction distance (see Figure 5C). This finding suggests that weakening and interruption of the D*X*Q homodimer halogen bonding interaction geometry likely results in the change in packing motif observed for DMDBS-DDQ compared to the remaining DMDB*Y*-D*X*Q cocrystals. Additionally, disruption of the homodimer halogen bonding interaction in the DMDBS-DDQ cocrystal is likely thermally accessible (dynamic) due to the relatively flat interaction enthalpy surface,^13^ providing a justification for the absence of whole-molecule disorder in all other DMDB*Y*-D*X*Q cocrystals despite high void volume. Interaction enthalpy surfaces for halogen bonding (in-plane interactions) were also compared to interaction enthalpy surfaces for charge-transfer along the π-stacking direction when paired with the DMDBS donor (see Figures 5D and SI4), supporting the claim that there is a significant difference in interaction enthalpy when comparing the in-plane and out-of-plane (π-stacking) directions of the cocrystals.

## 4 Conclusions

DMDBS-DDQ is a CT cocrystal system that presents an interesting case of non-isoreticularity and a high degree of disorder relative to other members of the DMDB*Y*-D*X*Q cocrystal family. Despite significant size mismatch between DMDBS and DDQ and the high degree of disorder in all cocrystals formed between DMDBS and DDQ, the DMDBS-DDQ cocrystal offers less void space than other cocrystals in the DMDB*Y*-D*X*Q cocrystal family. This finding is in direct opposition to previous design guidelines that claim that size-mismatch between CT coformers can yield high-void-space materials that facilitate dynamic functional disorder. Interaction enthalpy surfaces calculated for in-plane halogen bonds offer an alternative explanation: strong and directional in-plane interactions can rotationally lock molecules, yielding higher void space materials that do not demonstrate dynamic disorder. Despite this finding, the DMDBS-DDQ cocrystal system does suggest that targeting size mismatch in molecular coformers is a viable strategy toward designing dynamically disordered systems not because of an increase in void space encapsulation but instead because size-mismatched coformers are more likely to present an interaction polarizability mismatch (spatial mismatch in orbital overlap) that yields weaker and more flexible interaction geometries.

## Supplementary Material



## Notes

Experimental details; X-ray diffraction data; single-crystal X-ray structures; ^1^H NMR spectra; PXRD patterns; DSC traces; TGA traces; IR spectra; DFT calculations (PDF)
