# Supplementary material for: Design of Functional Disorder in Charge-Transfer Cocrystals
Source: Chem Mater. 2025 Oct 27;37(21):8944–51. doi: 10.1021/acs.chemmater.5c02166 (PMC12614039; doi:10.1021/acs.chemmater.5c02166)
Supplement: Supplementary file 1 [file cm5c02166_si_001.pdf]

Design of Functional Disorder in Charge-Transfer Cocrystals

Supplementary Information

Phoebe Eccles, Jesus Daniel Loya, Nina Aagaard, Abigail A. Moravek, and Ren A. Wiscons*

Department of Chemistry Amherst College, 25 East Dr, Amherst, MA, 01002, United States

e-mail: rwiscons@amherst.edu

Table of Contents:

SI 1. Experimental Information
SI 2. Crystallographic Data
SI 3. Cambridge Structural Database Survey of Disordered CT Cocrystal Structures
SI 4. Calculated PES, Corresponding Geometries, and Crystallographic Distances
SI 5. Analysis of Molecular Volumes and Crystallographic Void Spaces
SI 6. Di-*o*-tolyl selane (**1a**) and di-*o*-tolyl diselenide (**1b**) GCMS Chromatogram
SI 7. ^{1}H Nuclear Magnetic Resonance Spectra
SI 8. Powder X-ray Diffraction Patterns
SI 9. Differential Scanning Calorimetry Curve
SI 10. Thermogravimetric Analysis
SI 11. IR Spectra
SI 12. Polarization Hysteresis Loops
SI 13. Temperature-Dependent Lattice Constants for DMDBS-DDQ
SI 14. Oak Ridge Thermal Ellipsoid Plots (ORTEP) for SCXRD Structures
SI 15. References

SI 1. Experimental Information

All reagents were used as received from suppliers without additional purification. DDQ (2,3-dichloro-5,6-dicyano-1,4-benzoquinone), *o*-iodotoluene, copper (I) iodide (CuI), tribasic potassium phosphate (K_3PO_4), palladium (II) trifluoroacetate ($Pd^{II}(tfa)_2$), pivalic acid (PivOH), Se^0 powder, PEG_{500}, silver (I) acetate (AgOAc), sodium borohydride ($NaBH_4$), sodium *tert*-butoxide (NaO^tBu), anhydrous magnesium sulfate ($MgSO_4$), potassium carbonate (K_2CO_3), anhydrous methanol (MeOH), and dichloromethane (DCM) were obtained from Sigma Aldrich. Hexanes, tetrahydrofuran (THF), and acetonitrile were obtained from Alfa Aesar. Acetonitrile used for crystallization was dried over 3 Å molecular sieves for at least one day prior to use.

*1.1. Synthesis of di-o-tolyl selane (**1a**) and di-o-tolyl diselenide (**1b**)*

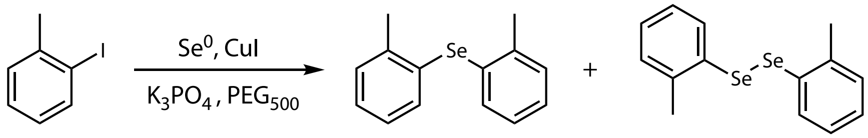

1a **1b**

Synthesis of 4,6-dimethyldibenzoselenophene (DMDBS) was adapted from previously reported literature procedure.[1] Se^0 powder (781.3 mg, 9.893 mmol) was reacted with *o*-iodotoluene (4335 mg, 19.88 mmol) in PEG_{500} (16 mL) in the presence of CuI (209.4 mg, 1.099 mmol) and tribasic K_3PO_4 (9,960 mg, 46.92 mmol) in a sealed 100 mL pear-shaped flask. The flask was heated to 125 °C in a silicone oil bath and stirred for 24 hours. The crude product was extracted with hexanes, washed with water, and dried over anhydrous $MgSO_4$. The material was concentrated to produce a yellow oil and purified further using $NaBH_4$ (*vide infra*). Selane intermediate **1a**: ^{1}H NMR (400 MHz, acetone-d_6) δ7.30 ppm (*d*, 2H), δ7.25 ppm (*t*, 2H), δ7.20 ppm (*d*, 2H), δ7.09 ppm (*t*, 2H), δ2.81 ppm (*s*, 6H). GCMS *o*-iodotoluene (retention time, rt 7.518 min, m/z 218), selane intermediate **1a** (rt 18.937 min, m/z 262), diselenide biproduct **1b** (rt 20.336, m/z 342).

*1.2. Purification of di-o-tolyl selane (**1a**) from di-o-tolyl diselenide (**1b**)*

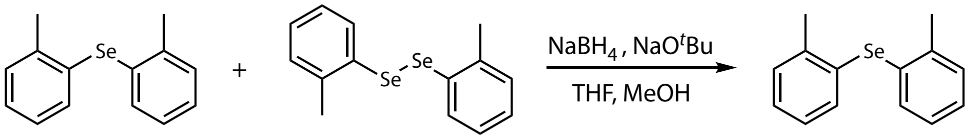

1a **1b** **1a**

The crude yellow oil from the previous synthetic step was mixed in a 100 mL round bottom flask with 2 molar equivalents of $NaBH_4$ and 1 molar equivalent of NaO^tBu in anhydrous methanol (10 mL) and THF (10 mL) under nitrogen atmosphere. The reaction was heated to 60 °C and stirred for 20 minutes. The mixture was swiftly washed *via* liquid-liquid extraction with hexanes and a solution of sodium thiosulfate and sodium hydroxide. The hexanes layer was dried over anhydrous $MgSO_4$ and concentrated under reduced pressure to a pink oil. The pink oil was purified by column chromatography on silica gel using a 10% (v/v) DCM in hexanes as the mobile phase. Column fractions were combined and concentrated to yield **1a** as a colorless oil.

1.3. *Synthesis of 4,6-dimethyldibenzoselenophene (**DMDBS**)*

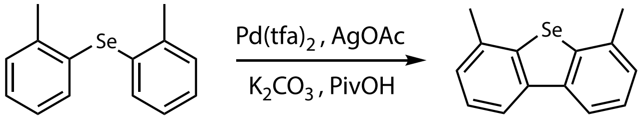

The selane intermediate **1a** (300.0 mg, 1.15 mmol) was reacted with K_2CO_3 (317.8 mg, 2.30 mmol), AgOAc (19.2 mg, 0.115 mmol), and $Pd(tfa)_2$ (192.3 mg, 0.575 mmol) in PivOH (2 mL) in a sealed 100 mL round bottom flask under nitrogen atmosphere. The reaction was stirred for 16 hours at 120 °C. The product was extracted into diethyl ether and filtered through a celite pad. It was then washed with a solution of sodium bicarbonate and dried over anhydrous $MgSO_4$. The crude product was purified by column chromatography on silica gel using a 10% (v/v) DCM in hexanes as the mobile phase. Column fractions were combined and concentrated to yield DMDBS. DMDBS: ^{1}H NMR (400 MHz, acetone-d_6) δ8.10 ppm (*d*, 2H), δ7.46 ppm (*t*, 2H), δ7.30 ppm (*d*, 2H), δ2.81 ppm (*s*, 6H). The NMR spectrum of the product matched the previously reported NMR spectra from the literature[1] and structure was further confirmed by single crystal X-ray diffraction.

1.4. *Crystallization of DMDBS$_X$-DDQ$_Y$*

A physical mixture of four DMDBS$_X$-DDQ$_Y$ cocrystal phases (DMDBS-DDQ, DMDBS-DDQ-ACN$_X$, DMDBS$_3$-DDQ$_2$, and DMDBS$_5$-DDQ$_4$) is produced through evaporative crystallization. DDQ (1 mg, 0.0044 mmol) and DMDBS (1 mg, 0.0039 mmol) were added to a 1.5 mL polypropylene centrifuge tube with 1.0 mL of acetonitrile, and sonicated to ensure full dissolution. The acetonitrile was slowly evaporated over a two-day period, yielding a mixture of DMDBS$_X$-DDQ$_Y$ cocrystal phases in the centrifuge tube.

1.5. *Crystallization of DMDBS-DDQ-ACN$_X$*

Single crystals of DMDBS-DDQ-ACN$_X$ were prepared by dissolving DDQ and DMDBS in acetonitrile. DDQ (1 mg, 0.0044 mmol) and DMDBS (1 mg, 0.0039 mmol) were added to a 1-dram vial with 0.5 mL of acetonitrile, and sonicated to ensure full dissolution. The vial was placed in a freezer (-18 °C) for 1 hour. Single crystals of DMDBS-DDQ-ACN$_X$ precipitated and the solvent was decanted.

1.6. *Desolvation of DMDBS-DDQ-ACN$_X$*

Crystals of DMDBS-DDQ-ACN$_X$ were dried under N_2 gas for 1 hour to ensure residual acetonitrile from the DMDBS-DDQ-ACN$_X$ crystallization was evaporated. Crystals of DMDBS-DDQ-ACN$_X$ were heated to 85°C under air or nitrogen atmosphere for 20 minutes to achieve DMDBS-DDQ.

1.7. *X-Ray Diffraction (XRD)*

1.7.a. Structural Determination by Single-Crystal X-ray Diffraction

X-ray diffraction images were collected using a Rigaku XtaLAB Synergy-i X-ray diffractometer configured in a kappa goniometer geometry. The diffractometer is equipped with a variable-temperature device and a PhotonJet-S microfocus Cu source (λ = 1.54187 Å) and operated at 50 kV and 1 mA. X-ray intensities were

measured at room temperature with the Bantam detector placed 44.00 mm from the sample. The data were processed with CrysAlisPro version 41_64.117a (Rigaku Oxford Diffraction) and corrected for absorption. The structures were determined in OLEX2[2] using SHELXT[3] and refined using SHELXL[4]. Except in cases of significant positional disorder, all non-hydrogen atoms were refined anisotropically with hydrogen atoms placed at idealized positions. Single crystals were mounted on a 150 µm MiTeGen MicroMount using mineral oil.

1.7.b. Powder X-ray Diffraction

X-ray diffraction images were collected using a Rigaku XtaLAB Synergy-i X-ray diffractometer configured in a kappa goniometer geometry. The diffractometer is equipped with a variable-temperature device and a PhotonJet-S microfocus Cu source (λ = 1.54187 Å) and operated at 50 kV and 1 mA. X-ray intensities were measured at between 100 and 350 K with the Bantam detector placed 50.00 mm from the sample. All images were collected with a continuous ϕ-rotation scan with a 200 second exposure time. The images were integrated and processed into diffraction patterns using CrysAlisPro version 41_64.117a (Rigaku Oxford Diffraction). Samples were mounted on a 100 µm MiTeGen MicroMount using mineral oil. This data collection strategy minimized the effects of preferred orientation.

1.7.c. Measurement of DMDBS-DDQ Lattice Constants

Lattice constants were measured from X-ray diffraction images collected using a Rigaku XtaLAB Synergy-i X-ray diffractometer configured in a kappa goniometer geometry. The diffractometer is equipped with a variable-temperature device and a PhotonJet-S microfocus Cu source (λ = 1.54187 Å) and operated at 50 kV and 1 mA. X-ray intensities were measured at between 100 and 350 K with the Bantam detector placed 44.00 mm from the sample. For each measurement of the lattice parameters, the data collection strategy was designed to achieve 60% completeness (for a full *P*-1 SCXRD collection), an I/σ of 10, and a redundancy of 3.

1.8. Nuclear Magnetic Resonance (NMR)

^{1}H NMR spectra were collected on a Bruker Ascend 400 MHz spectrometer operating at room temperature. For each spectrum, 16 scans and a 10 second relaxation delay were used. Samples were prepared by dissolution in acetone-d_6. Chemical shifts and signal integrations were determined using MestReNova v14.2.2-28739, MestReLab Research, 2021.

1.9. Differential Scanning Calorimetry (DSC)

Differential scanning calorimetry (DSC) traces were measured on a Mettler Toledo Instrument DSC 3^{+} equipped with a Hubber TC100 cooling system under nitrogen atmosphere (50 mL/min). All experiments were run in hermetic aluminum DSC pans with a heating rate of either 5 °C/min or 10 °C/min, covering a temperature range of 25 °C to 175 °C. DSC traces were analyzed using METTLER STARe default data viewer.

1.10. Thermogravimetric Analysis (TGA)

TGA experiments were performed on a Mettler Toledo instrument TGA 2, equipped with a Hubber mini chiller 300 cooling system, under nitrogen atmosphere (20 mL/min) with an aluminum pan at a rate of 10 °C/min. TGA traces were analyzed using METTLER STARe default data viewer.

1.11. Fourier Transform Infrared (FT-IR) Spectroscopy

FTIR spectra were obtained using a Bruker Tensor 37 instrument equipped with an MIRacle™ single reflection Attenuated Total Reflectance (ATR) accessory with a diamond/ZnSe window from PIKE Technologies. For each spectrum, 32 scans of the background and sample were collected. Background subtractions were performed using OPUS v6.5 Build 6, 5, 92 (Bruker Optik).

1.12. Gas Chromatography Mass Spectrometry (GCMS)

GCMS data were collected using an Agilent Technologies 7890A GC system equipped with a (5%-phenyl) methylpolysiloxane non-polar column coupled to a mass spectrometer with an EI ionization source. GCMS samples were prepared by filtration through silica (when necessary) and dilution with DCM. Samples were run between 80 to 180 °C over 18 min then heated to 275 °C over 5 min. Total ion chromatograms and mass spectra were processed using MassHunter (Agilent Technologies) and OpenChrom Eclipse using the MassHunter plug-in.

1.13. Electrical Property Characterization.

1.13.a. Device Fabrication
Single-crystal devices of desolvated DMDBS-DDQ cocrystals were prepared by immobilizing crystals on a 1 cm x 1 cm glass slide with minimal vacuum grease. Using the crystal face indexation package available in CrysAlis Pro (Rigaku), silver contacts (Dupont Micromax™ 4922N) were placed on opposite (001) faces of the immobilized DMDBS-DDQ single crystals using 50 μm diameter aluminum wire to make a two-point capacitance measurement.

1.13.b. Electronic Characterization
Polarization hysteresis loops were collected using a Precision Multiferroic II Test System (Radiant Technologies, Inc.) using Vision Software and a variable-temperature four-point probe station (Linkam Scientific). Polarization hysteresis loops were collected between 22 °C and 75 °C and all presented polarization hysteresis loops were collected between +/-25 V and at 2 Hz. Device dimensions were used to convert the measured capacitance into net polarization and the drive voltage into electric field.

1.14. Computational Methods

1.14.a. Molecular Properties
Single point energy calculations at ground state in the gas phase were performed using Spartan '20. Atom positions were obtained from single-crystal X-ray diffraction data. DFT was used with the ωB97X-D exchange-correlation functional and the split valance basis set 6-31G(*). Electrostatic potential maps and density surfaces were computed and mapped using the isodensity surface 0.002 e/au^3 with medium resolution.

1.14.b. Interaction Enthalpy Surfaces

Potential energy surfaces (PESs) of D*X*Q-D*X*Q (i.e., DDQ, DBQ, DIQ) homodimers and D*X*Q-DMDBS heterodimers were calculated by loading a halogen bonded or π-stacked dimer onto Spartan ’20. The geometric coordinates of the atom positions of the dimers were obtained from single-crystal X-ray diffraction data. The distances of the halogen bond (homodimers) or the π- π interactions (heterodimers) were systematically varied and the energy at each point was computed with the ωB97X-D exchange-correlation functional and the split valance basis set 6-31G(*).

1.14.c. Crystallographic Void Space Determination

Crystallographic void space was measured to quantify the packing density of the cocrystals. This was performed using the visualization and analysis software Mercury from the Cambridge Crystallographic Data Centre (CCDC).[5] The Pore Analyser tool was used with a probe radius of 0.53 Å and grid spacing of 0.3 Å to obtain the void volume of the crystal structures. The void volume was converted into the percent void volume per unit cell by dividing the void volume by the room-temperature unit cell volume.

SI 2. Crystallographic Data

Table SI 1. Table of Crystallographic Parameters

Compound name	DMDBS-II	DMDBS-DDQ-ACN*x*	DMDBS-DDQ	$DMDBS_3$-DDQ_2	$DMDBS_5$-DDQ_4
CCDC Refcode	2464758	2464756	2464757	2464760	2464759
Chemical formula	$C_{14}H_{12}Se$	$C_{14}H_{12}Se{\cdot}C_8Cl_2N_2O_2{\cdot}0.33(C_2H_3N)$	$C_{14}H_{12}Se{\cdot}C_8Cl_2N_2O_2$	$3(C_{14}H_{12}Se){\cdot}2(C_8Cl_2N_2O_2)$	$5(C_{14}H_{12}Se){\cdot}4(C_8Cl_2N_2O_2)$
M_r	259.20	499.74	486.20	1231.59	2203.98
Crystal system	Monoclinic	Triclinic	Triclinic	Monoclinic	Triclinic
Space group	$P2_1/c$	*P*-1	*P*-1	*C*2/*m*	*P*-1
Temperature (K)	150	294	293	294	294
a (Å)	9.6660 (3)	6.6275 (5)	6.6159 (8)	21.2225 (13)	9.8294 (2)
B (Å)	6.6103 (2)	9.8639 (6)	9.6710 (18)	18.1751 (11)	14.1383 (4)
c (Å)	16.8504 (5)	17.311 (1)	16.6121 (15)	6.6078 (4)	17.7074 (4)
α (°)	90	79.413 (5)	77.021 (12)	90	97.445 (2)
β (°)	90.569 (3)	80.927 (5)	88.849 (9)	93.725 (5)	105.129 (2)
γ (°)	90	71.117 (6)	72.454 (13)	90	104.982 (2)
V ($Å^3$)	1076.61 (6)	1046.65 (12)	986.2 (2)	2543.4 (3)	2242.94 (10)
Z	4	2	2	2	1
Radiation type	Cu *K*α	Cu *K*α	Cu *K*α	Cu *K*α	Cu *K*α
μ (mm^{-1})	4.37	4.97	5.26	5.00	5.15
Crystal size (mm)	0.38 × 0.19 × 0.03	0.25 × 0.03 × 0.02	0.17 × 0.03 × 0.02	0.22 × 0.03 × 0.02	0.19 × 0.13 × 0.08
Data collection					
Diffractometer	XtaLAB Synergy-S	XtaLAB Synergy-S	XtaLAB Synergy-S	XtaLAB Synergy-S	XtaLAB Synergy-S
Absorption correction	Multi-scan	Multi-scan	Multi-scan	Multi-scan	Multi-scan
T_{min}, T_{max}	0.612, 1.000	0.875, 1.000	0.908, 1.000	0.931, 1.000	0.748, 1.000
No. measured reflections	3257	5165	4016	2782	18267
No. independent reflections	1641	2519	1995	1330	8028
No. observed [$I > 2\sigma(I)$] reflections	1470	1996	1455	1097	6764
R_{int}	0.038	0.043	0.029	0.029	0.035
$(\sin \theta/\lambda)_{max}$ ($Å^{-1}$)	0.601	0.528	0.527	0.500	0.602
Refinement					
$R[F^2 > 2\sigma(F^2)]$, $wR(F^2)$, S	0.038, 0.104, 1.06	0.095, 0.286, 1.12	0.083, 0.244, 1.00	0.063, 0.185, 1.11	0.065, 0.176, 1.19
No. of reflections	1641	2519	1995	1330	8028
No. of parameters	139	263	259	169	664
No. of restraints	0	3	2	0	0
$\Delta\rho_{max}$, $\Delta\rho_{min}$ (e $Å^{-3}$)	0.41, -0.74	2.44, -0.63	1.33, −1.03	1.29, −0.56	0.57, -0.61

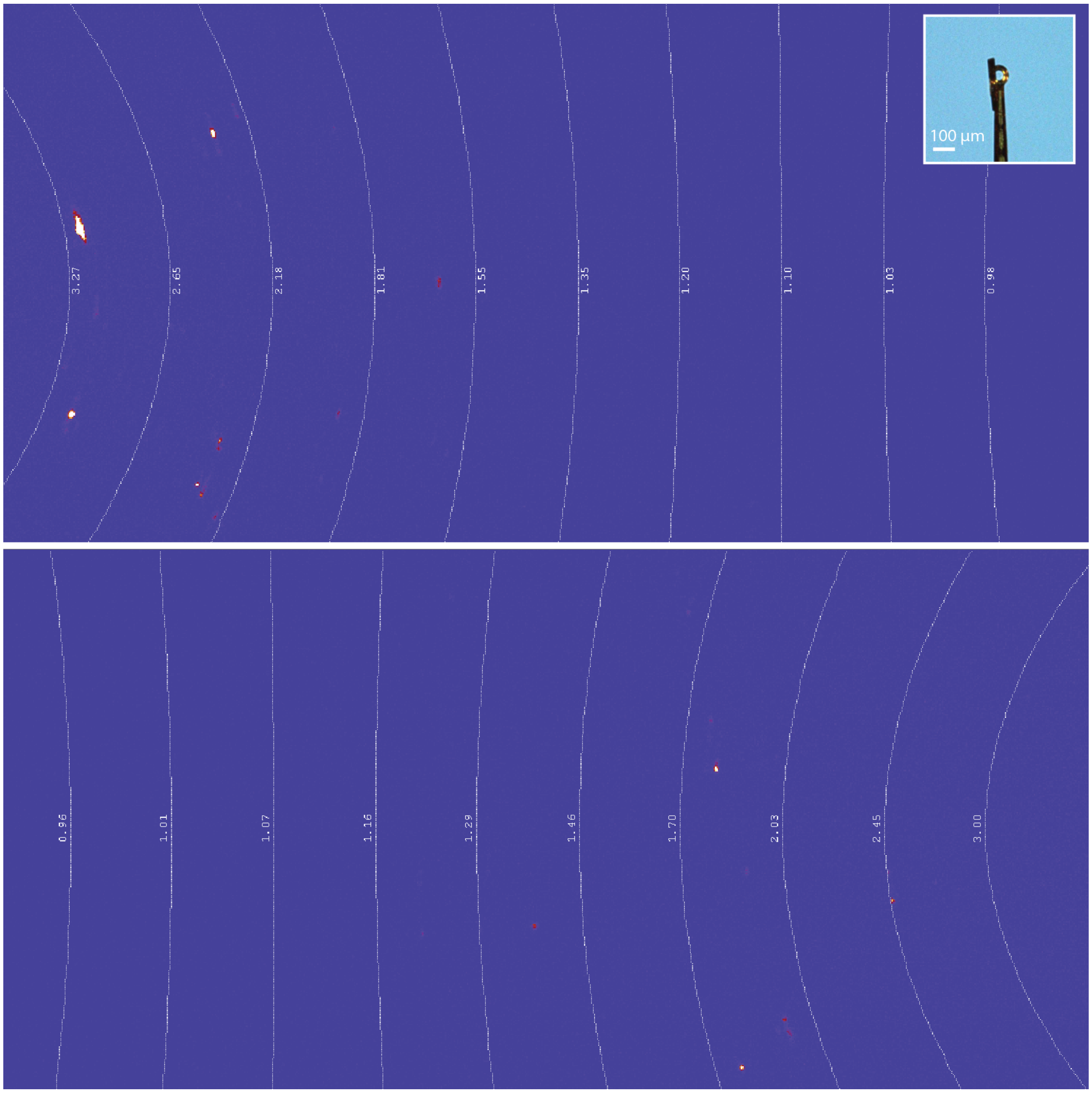

Figure SI 1. Representative diffraction images of the DMDBS-DDQ-ACN$_X$ cocrystal solvate and image of the diffracted crystal.

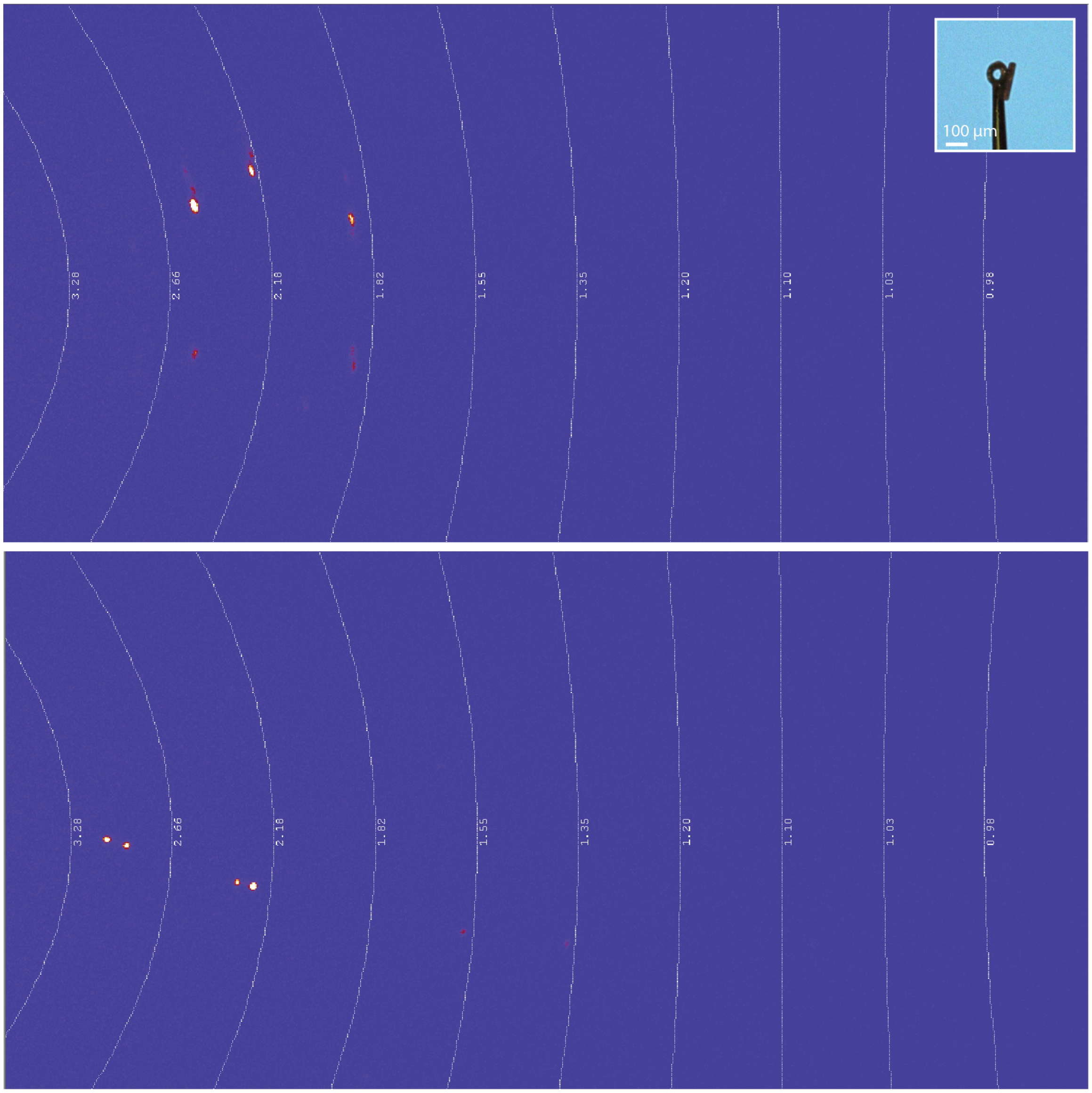

Figure SI 2. Representative diffraction images of the desolvated DMDBS-DDQ cocrystal and image of the diffracted crystal.

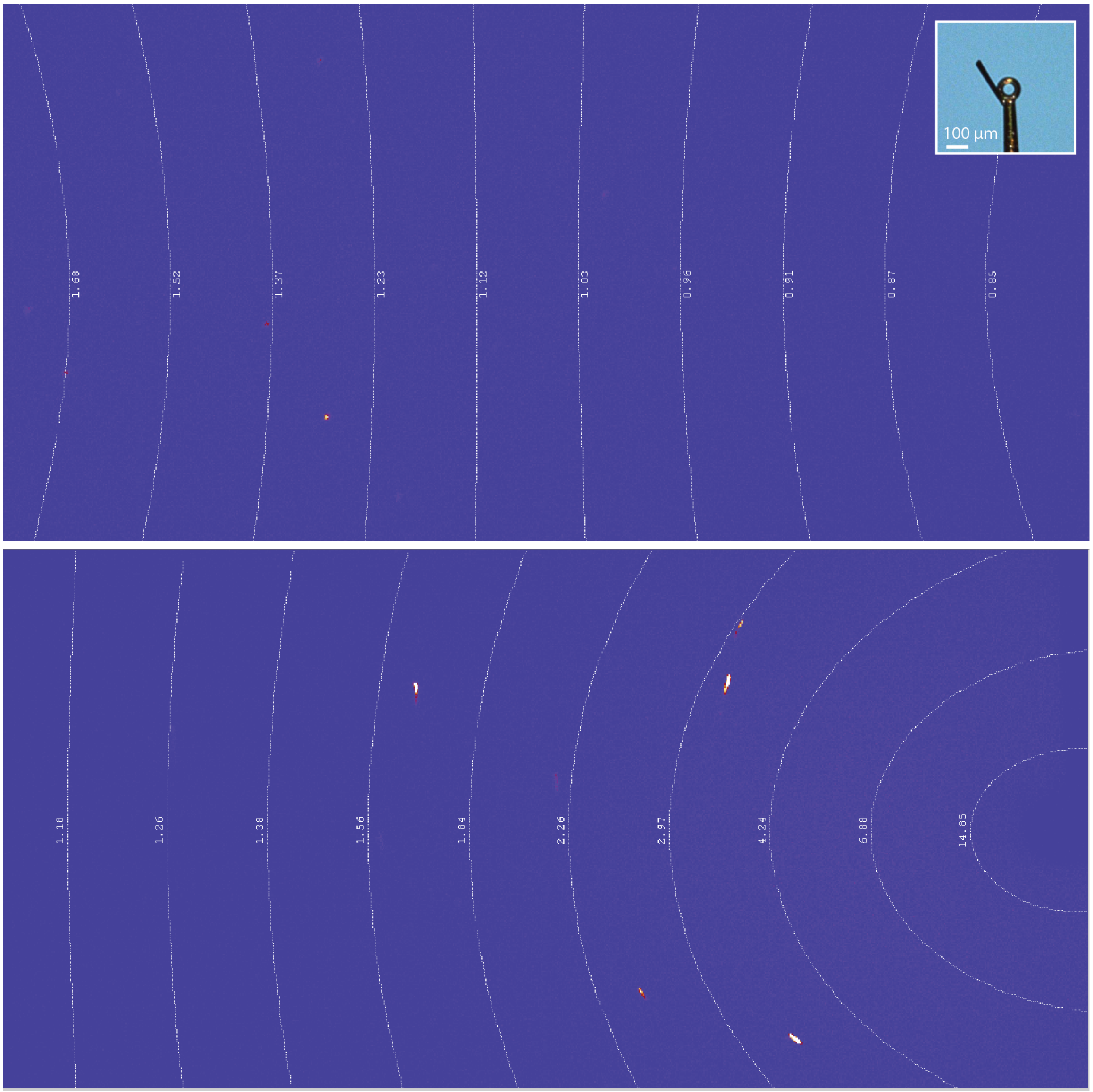

Figure SI 3. Representative diffraction images of the $DMDBS_3$-DDQ_2 cocrystal and image of the diffracted crystal.

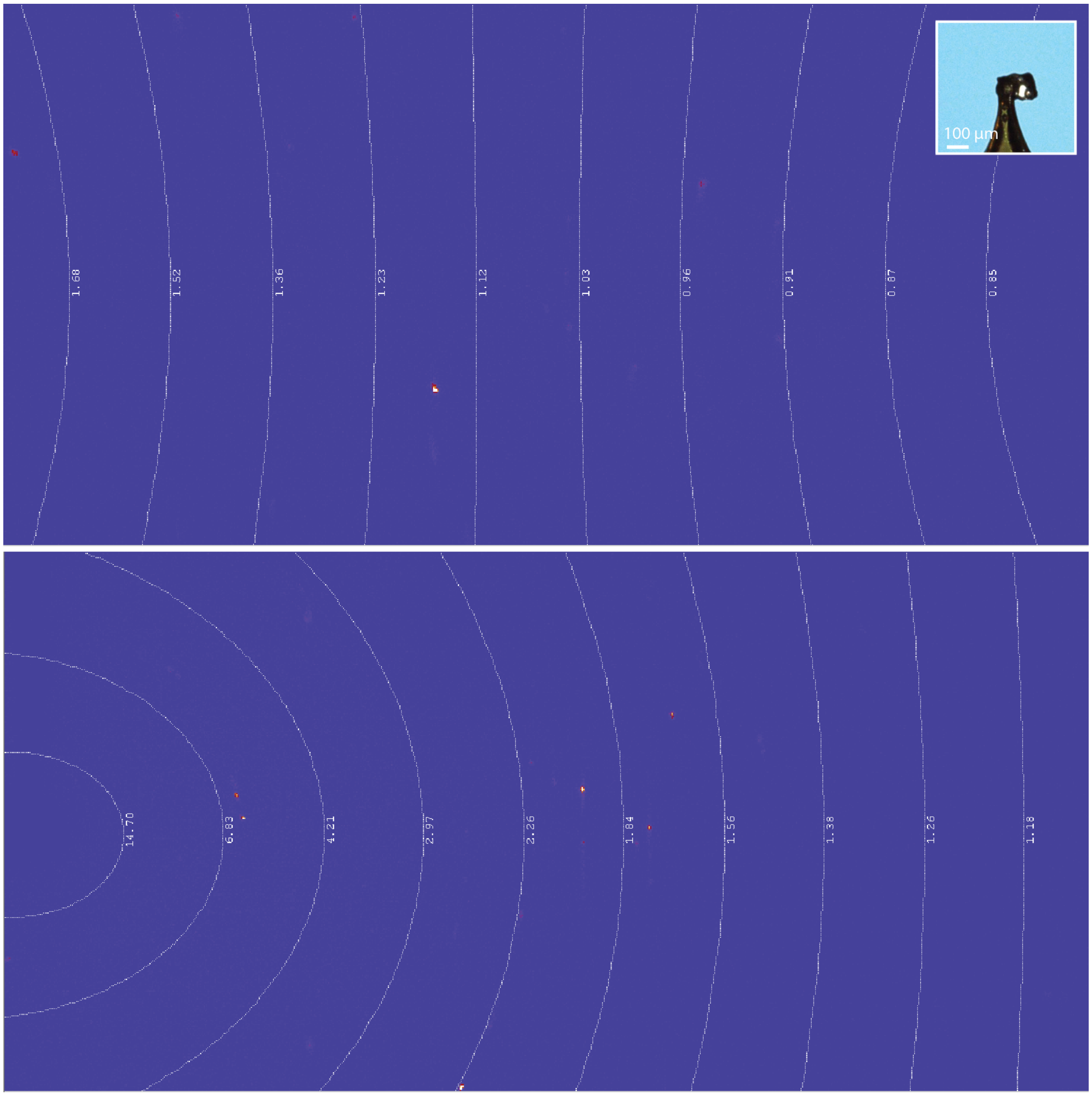

Figure SI 4. Representative diffraction images of the $DMDBS_5$-DDQ_4 cocrystal and image of the diffracted crystal.

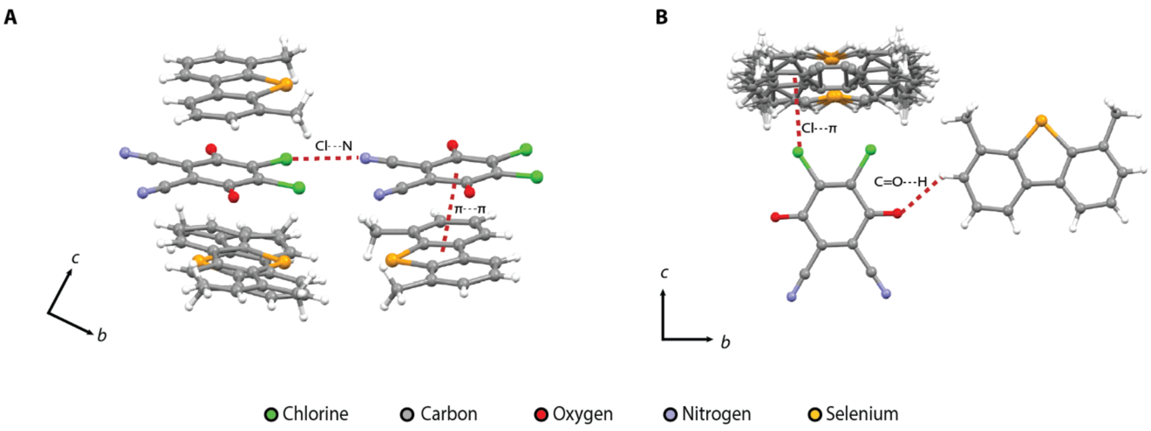

Figure SI 5. A) $DMDBS_3$-DDQ_2 packs in a 3:2 ratio of DMDBS to DDQ, and it crystallizes in the centro-symmetric C_2/m monoclinic space group. There are three crystallographically unique molecules of DMDBS, one of which is positionally disordered over two sites (site occupancy: 0.5:0.5). The cocrystal is sustained via π···π interactions between alternating DMDBS and DDQ molecules, which form into sheets. In addition to the π···π interactions, there are Cl···NC halogen bonds intermolecularly connecting adjacent DDQ molecules. **B)** $DMDBS_5$-DDQ_4 crystallizes in the triclinic centrosymmetric space group *P*-1. This structure is composed of five unique DMDBS molecules and 4 DDQ molecules. The DMDBS is both positionally disordered and lies on an inversion center. Much like $DMDBS_3$-DDQ_2 the crystal structure is sustained through π···π interactions between DMDBS and DDQ molecules, with the key exception of no halogen bonding occurring between DDQ molecules. Instead of halogen bonding there is Cl···π interactions that stop the halogen bonding from occurring and locking the DDQ molecule in place.

SI 3. Cambridge Structural Database Survey of Disordered CT Cocrystal Structures

The Structure Search feature on the Cambridge Crystallographic Data Centre's (CCDC) WebCSD portal was used to filter Cambridge Structural Database (CSD) entries by similarity to the skeletal structure of neutral quinoidal DDQ, DCQ (2,3-dicyano-1,4-benzoquinone), DBQ (2,3-dibromo-5,6-dicyano-1,4-benzoquinone), DIQ (2,3-diiodo-5,6-dicyano-1,4-benzoquinone), or TCPN (tetrachlorophthalonitrile). The results of these initial searches were then filtered to include only neutral charge-transfer cocrystals, which were then sorted by whether disorder was modeled in the CSD entry and visually inspected for disorder of the CT acceptor. The results of these searches are provided below.

Table SI 2. WebCSD Results

CSD Refcode	Details:	Notes:
DDQ Results	6 entries / 27 entries, 22%	
BZPCBQ		
FOXCAZ	Disorder	Disorder of DDQ over two positions related by 180° rotation
FOXDAA		
IHEMOB		
KUQXIG		
MEXVIW	Disorder	Disorder of DDQ over two positions related by 180° rotation
MUFZEU	Disorder	Disorder of DDQ over two positions related by 180° rotation
PANCYQ		
PEFCEI		
PEMBEE		
PIGNIC	Disorder	Disorder of DDQ over two positions related by 180° rotation
QATBEU	Solvated	Acetonitrile trapped in the structure
QIHXOX		
SOQKAN		
SOXJIY		
TEJGUK		
TEPMUW		
VEDYOU		
VEDYUA		
XEZHOD		
XEZHUJ		
XEZJAR		
XEZJEV	Disorder	Disorder of DDQ over two positions related by 180° rotation
XEZJIZ		
XEZJOF	Disorder	Disorder of carbazole over two positions related by 180° rotation
XOLCAE	Disorder and Solvated	Disorder of DDQ over two positions related by 180° rotation;
ZPHCYQ		
DCQ Results	0 entries / 4 entries, 0%	
KUQWOL		
KUQXOM		
KUQYED		
KUQYON	Disorder	Disorder of selenium atom on donor molecule
DBQ Results	0 entries / 6 entries, 0%	
FOXBUS		
FOXCUT		
FOXCUT01		
KUQXAY		
KUQXUS		
KUQYAZ		
DIQ Results	0 entries / 4 entries, 0%	
KUQWIF		
KUQWUR		
KUQYUT		
KUQZAA		

Table 2 continues on next page

Table SI 2. WebCSD Results (*continued*)

TCPN Results	5 entries / 7 entries (71%)	
EKATET	Disorder	Disorder of TCPN
GOHYUZ		
JIYTUJ	Disorder	Disorder of TCPN over two positions related by 60° rotation
JIYVEY	Disorder	Disorder of TCPN over two positions related by 60° rotation
JIYVOF	Disorder	Disorder of TCPN over two positions related by 120° rotation
MOCCEM		
PINJUU	Disorder	Disorder of TCPN

SI 4. Calculated PES, Corresponding Geometries, and Crystallographic Distances

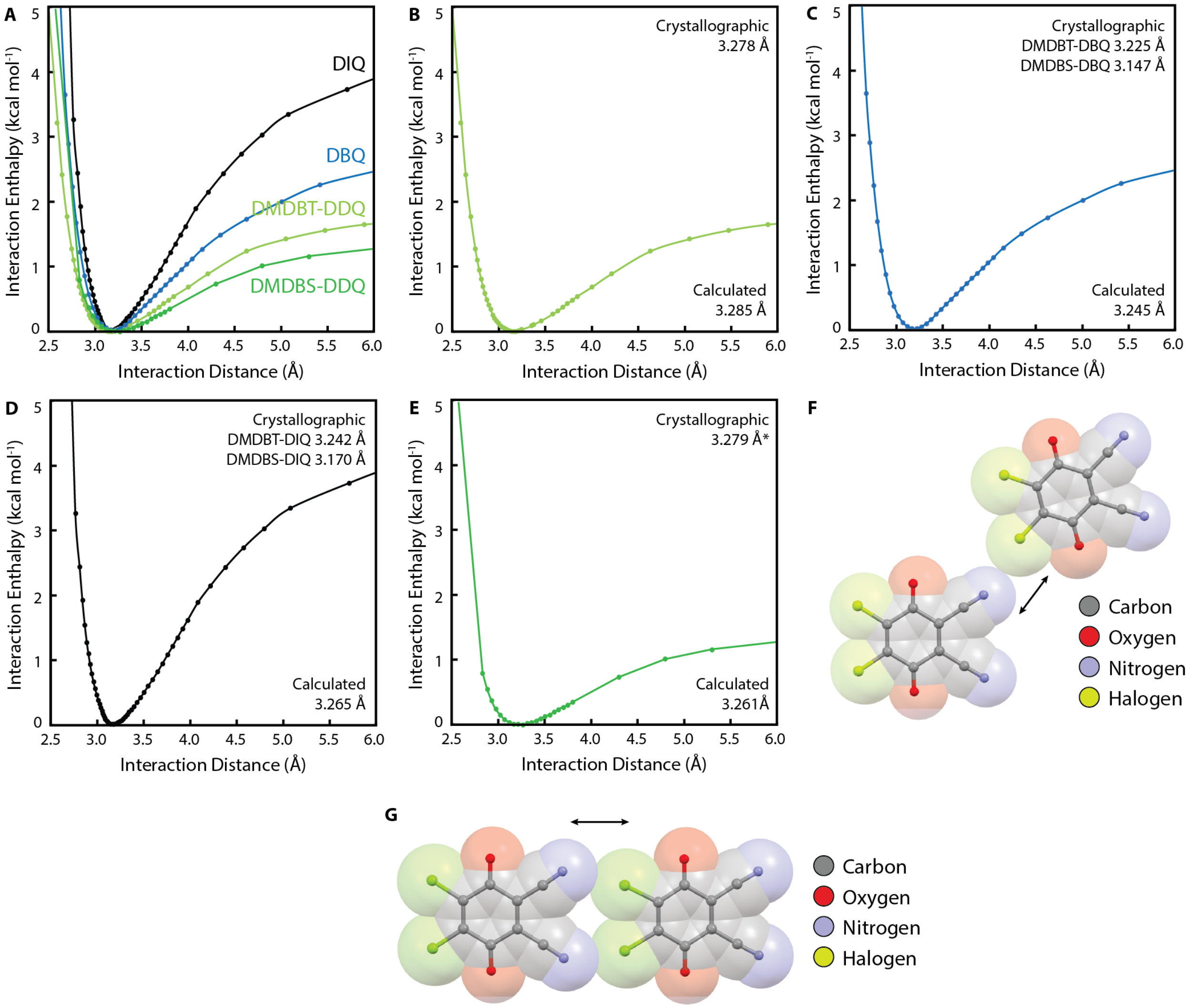

Figure SI 6. A) Summary of all interaction enthalpy surfaces for halogen bonding between homodimers either in the $Cmc2_1$ interaction geometry (DMDBT-DDQ, DBQ, DIQ) or in the *P*-1 interaction geometry (DMDBS-DDQ). **B)** Interaction enthalpy surface for a DDQ homodimer halogen bond in the $Cmc2_1$ interaction geometry. **C)** Interaction enthalpy surface for a DBQ homodimer halogen bond in the $Cmc2_1$ interaction geometry. **D)** Interaction enthalpy surface for a DIQ homodimer halogen bond in the $Cmc2_1$ interaction geometry. **E)** Interaction enthalpy surface for a DDQ homodimer halogen bond in the *P*-1 interaction geometry. The measured crystallographic interaction distances are given as well as the calculated minimum energy interaction distances. *This distance represents an average of the two unique crystallographic Cl···Cl distances present in the *P*-1 DDQ homodimer. **F)** Representation of the interaction geometry calculated for each $Cmc2_1$ homodimer with the double-headed arrow demonstrating the vector along which the interaction distance was changed to calculated the homodimer PESs. **G)** Representation of the interaction geometry calculated for the *P*-1 homodimer with the double-headed arrow demonstrating the vector along which the interaction distance was changed to calculate the PES.

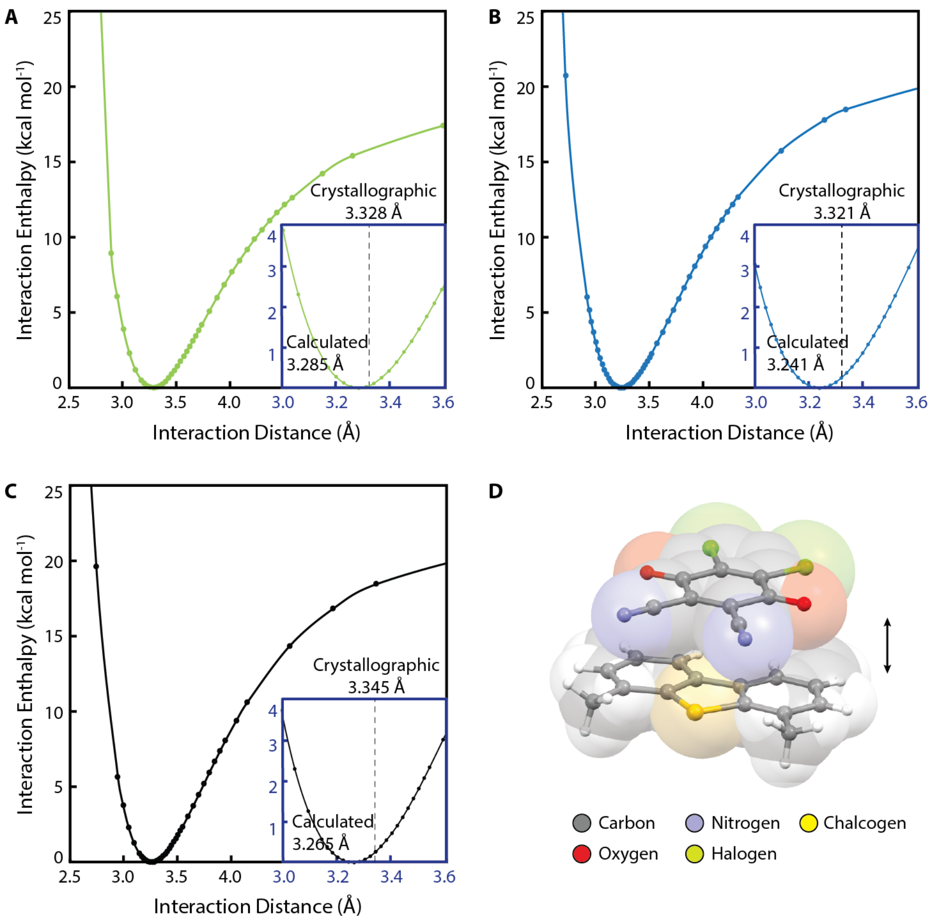

Figure SI 7. Interaction enthalpy surface for a charge-transfer heterodimer between DMDBS and **A)** DDQ, **B)** DBQ, or **C)** DIQ. The measured crystallographic interaction distances (room temperature) are given as vertical dashed lines in each inset, as well as the calculated minimum energy interaction distances. **D)** Generic representation of the interaction geometry calculated for each heterodimer with the double-headed arrow demonstrating the vector along which the interaction distance was changed to calculate the heterodimer PESs.

SI 5. Analysis of Molecular Volumes and Crystallographic Void Spaces

Table SI 3. Table of Molecular Volumes and Crystallographic Void Spaces

Cocrystal	DMDB*Y* Volume (Å^3)	D*X*Q Volume (Å^3)	Volume Ratio (D*X*Q/DMDB*Y*)	Void Volume (Å^3)	Void Space (%)
$DMDBS_3$-DDQ_2	230.0	182.2	0.7919	284.79	11.20
$DMDBS_5$-DDQ_4	230.0	182.2	0.7919	267.10	11.90
DMDBS-DDQ	230.0	182.2	0.7919	246.12	12.40
DMDBT-DDQ	225.6	182.2	0.8075	275.94	13.80
DMDBT-DIQ	225.6	213.3	0.9455	307.41	14.50
DMDBT-DBQ	225.6	193.4	0.8573	298.56	14.60
DMDBS-DBQ	230.0	193.4	0.8409	315.69	15.20
DMDBS-DIQ	230.0	213.5	0.9283	346.79	16.00

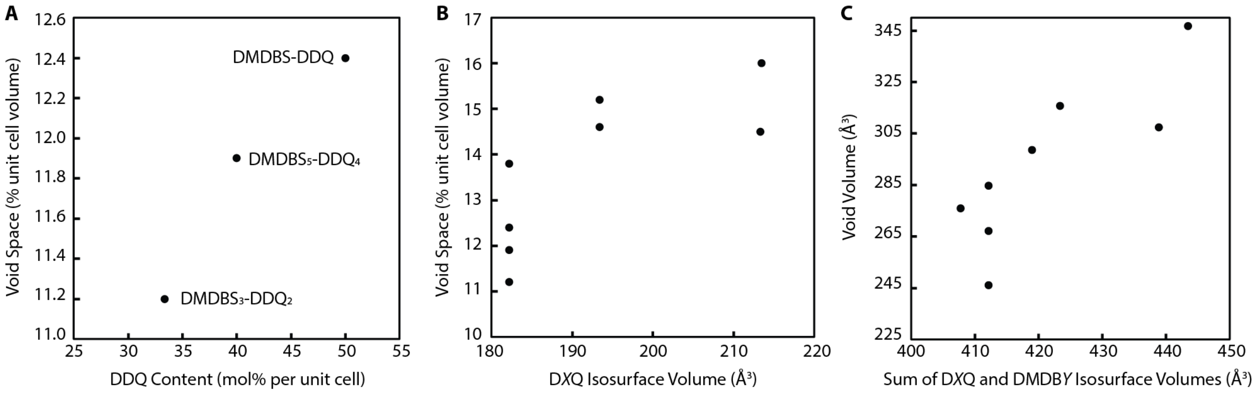

Figure SI 8. A) Plot showing the correlation between mol% of DDQ in the unit cell and calculated percent void space in the unit cell; **B)** plot of the volume enclosed by the D*X*Q and the calculated percent void space in the unit cell; **C)** relationship between the sum of the D*X*Q and DMDB*Y* isosurface volumes and the calculated void space volume.

SI 6. Di-*o*-tolyl selane (1a) and di-*o*-tolyl diselenide (1b) GCMS Chromatogram

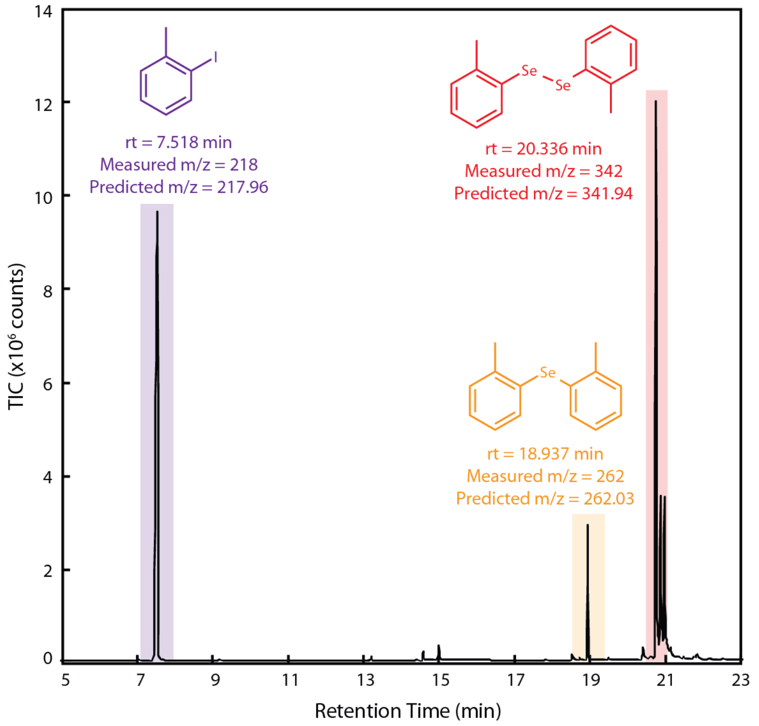

Figure SI 9. GCMS chromatogram of **1a**, di-*o*-tolyl selane, and **1b**, di-*o*-tolyl diselenide, in DCM.

SI 7. ^{1}H Nuclear Magnetic Resonance Spectra

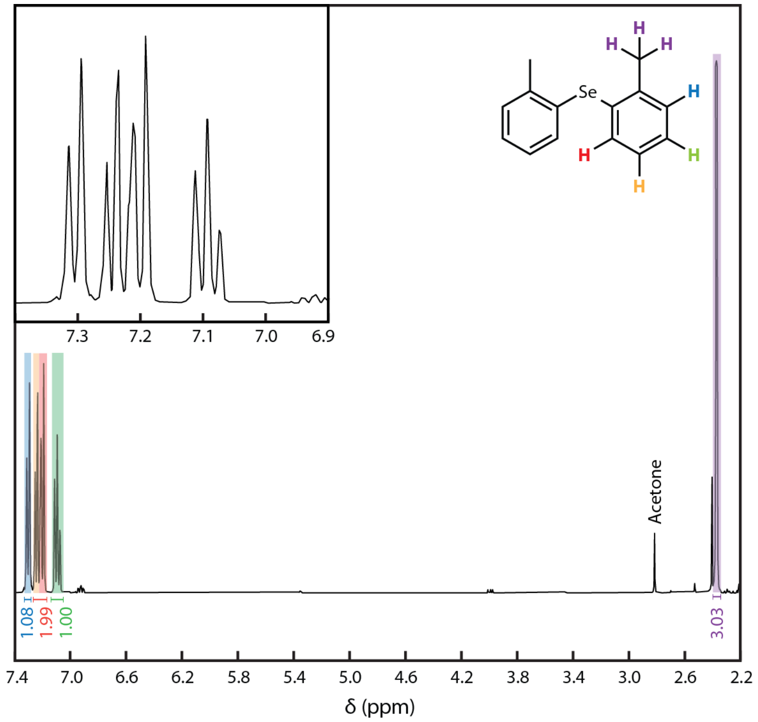

Figure SI 10. ^{1}H NMR spectrum of **1a**, di-*o*-tolyl selane intermediate, in acetone-d_6.

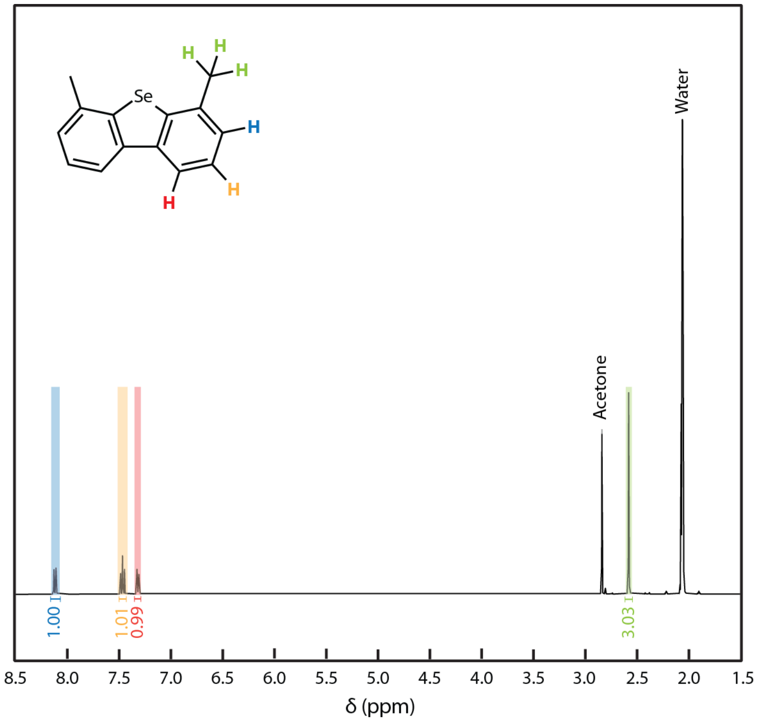

Figure SI 11. ^{1}H NMR spectrum of **DMDBS**, 4,6-dimethyldibenzoselenophene, in acetone-d_6.

SI 8. Powder X-Ray Diffraction Patterns

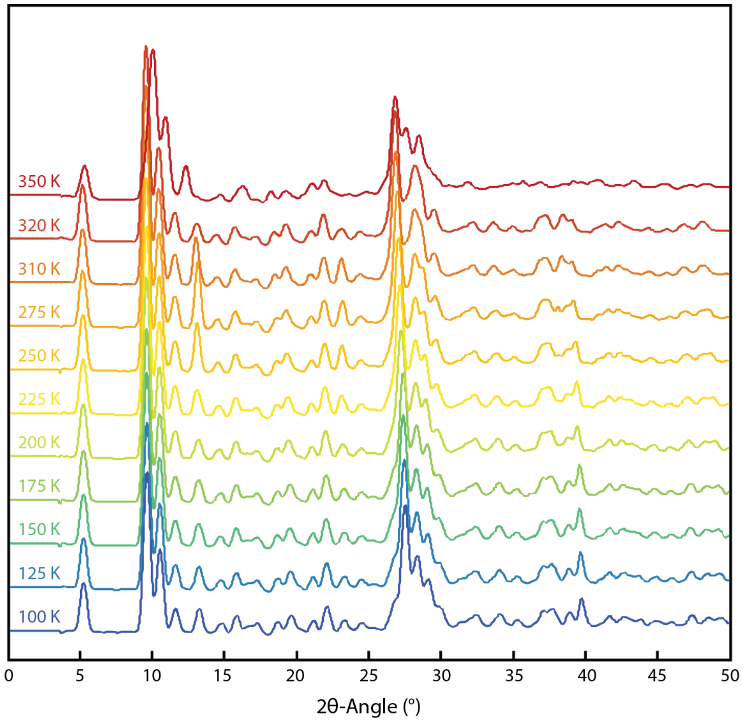

Figure SI 12. Powder X-ray diffraction patterns measured for DMDBS-DDQ-ACN$_X$ between 100 and 350 K. The temperature at which each diffraction pattern was measured is given along the left axis. All diffraction patterns were collected upon warming from 100 K.

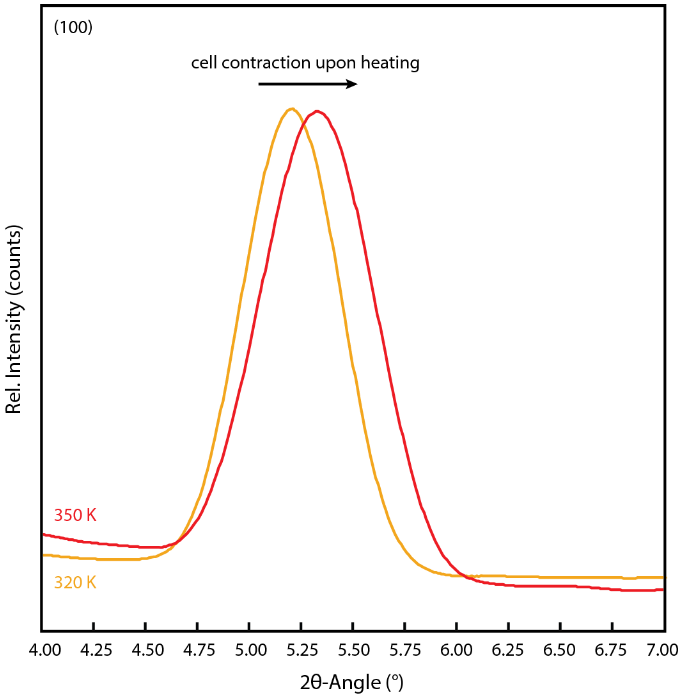

Figure SI 13. Powder X-ray diffraction patterns measured for DMDBS-DDQ-ACN$_X$ at 320 and 350 K (upon warming) centered on the (100) peak at ~5° 2θ, demonstrating contraction of the lattice upon heating. This behavior is consistent with desolvation of acetonitrile from DMDBS-DDQ-ACN$_X$ upon heating to 77 °C (350 K) to generate the DMDBS-DDQ cocrystal.

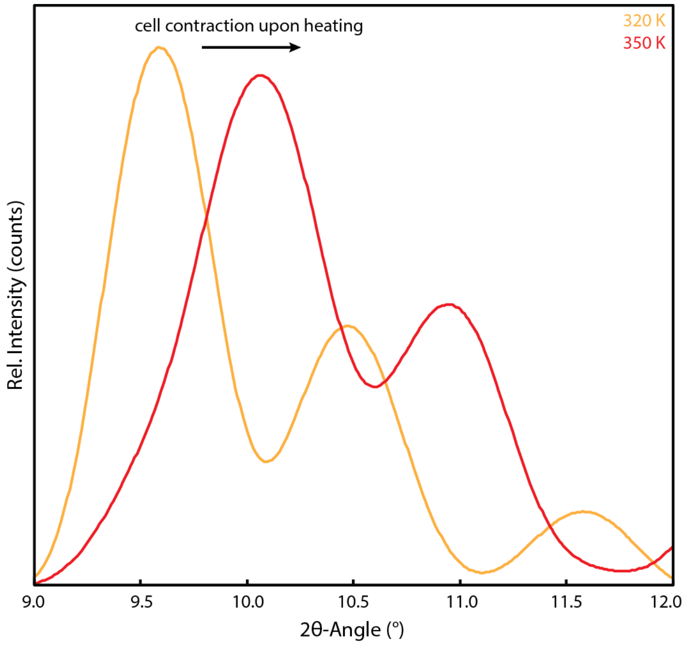

Figure SI 14. Powder X-ray diffraction patterns measured for DMDBS-DDQ-ACN$_X$ at 320 and 350 K (upon warming) centered on the (010) peak at ~10° 2θ, demonstrating contraction of the lattice upon heating. This behavior is consistent with desolvation of acetonitrile from DMDBS-DDQ-ACN$_X$ upon heating to 77 °C (350 K) to generate the DMDBS-DDQ cocrystal.

SI 9. Differential Scanning Calorimetry Curve

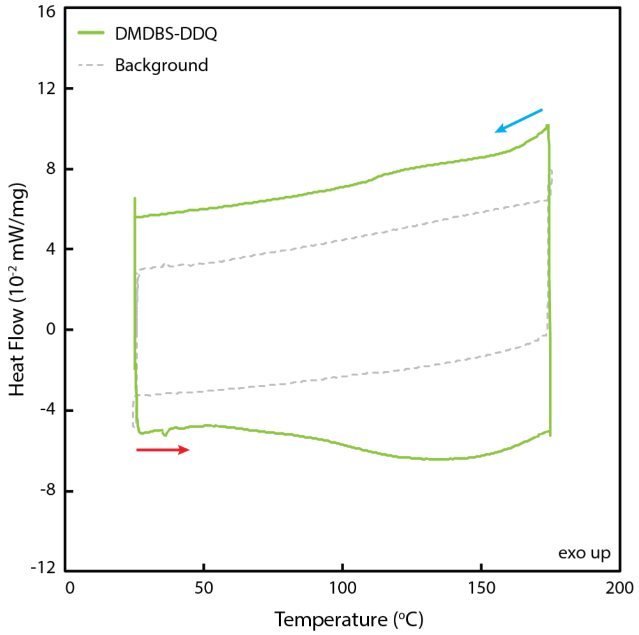

Figure SI 15. DSC curve of desolvated DMDBS-DDQ (solid green) and an empty aluminum DSC pan for reference (dashed gray) heated to 175 °C and cooled to 25 °C (10°C/min heating rate).

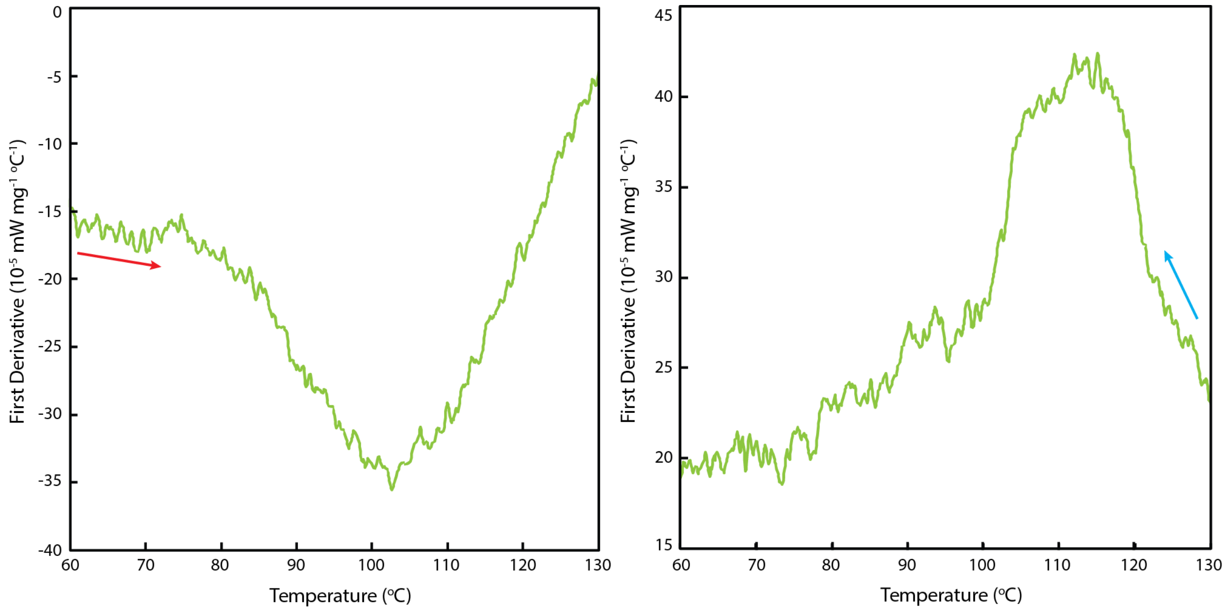

Figure SI 16. First derivative of the DMDBS-DDQ DSC curve upon heating (left) and upon cooling (right).

SI 10. Thermogravimetric Analysis

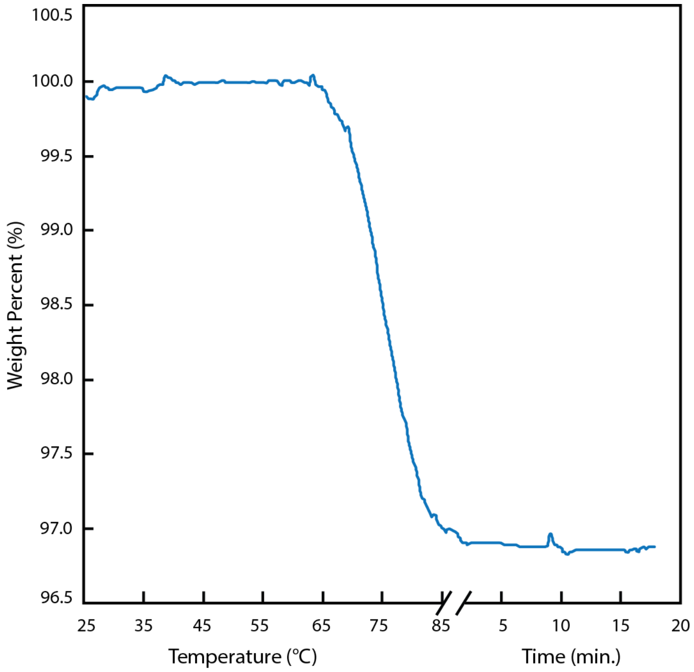

Figure SI 17. TGA curve of DMDBS-DDQ-CH_3CN_X (heating at 10°C/min and holding at 85°C for 20 minutes).

SI 11. IR Spectra

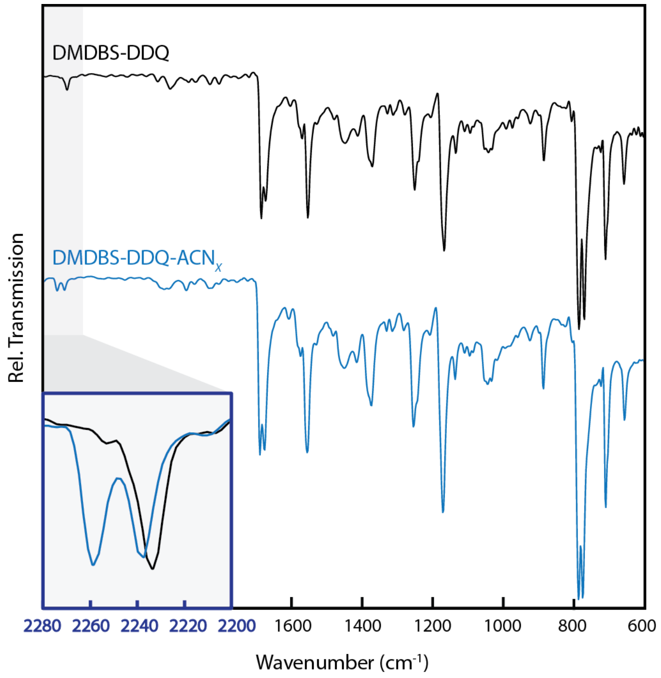

Figure SI 18. IR spectra of DMDBS-DDQ-ACN$_X$ taken before (blue) and after (black) the desolvation via TGA. Inset shows the disappearance of a nitrile peak upon desolvation attributed to loss of acetonitrile.

SI 12. Polarization Hysteresis Loops

12.1. Variable-Temperature Polarization Hysteresis Loops for Device 1

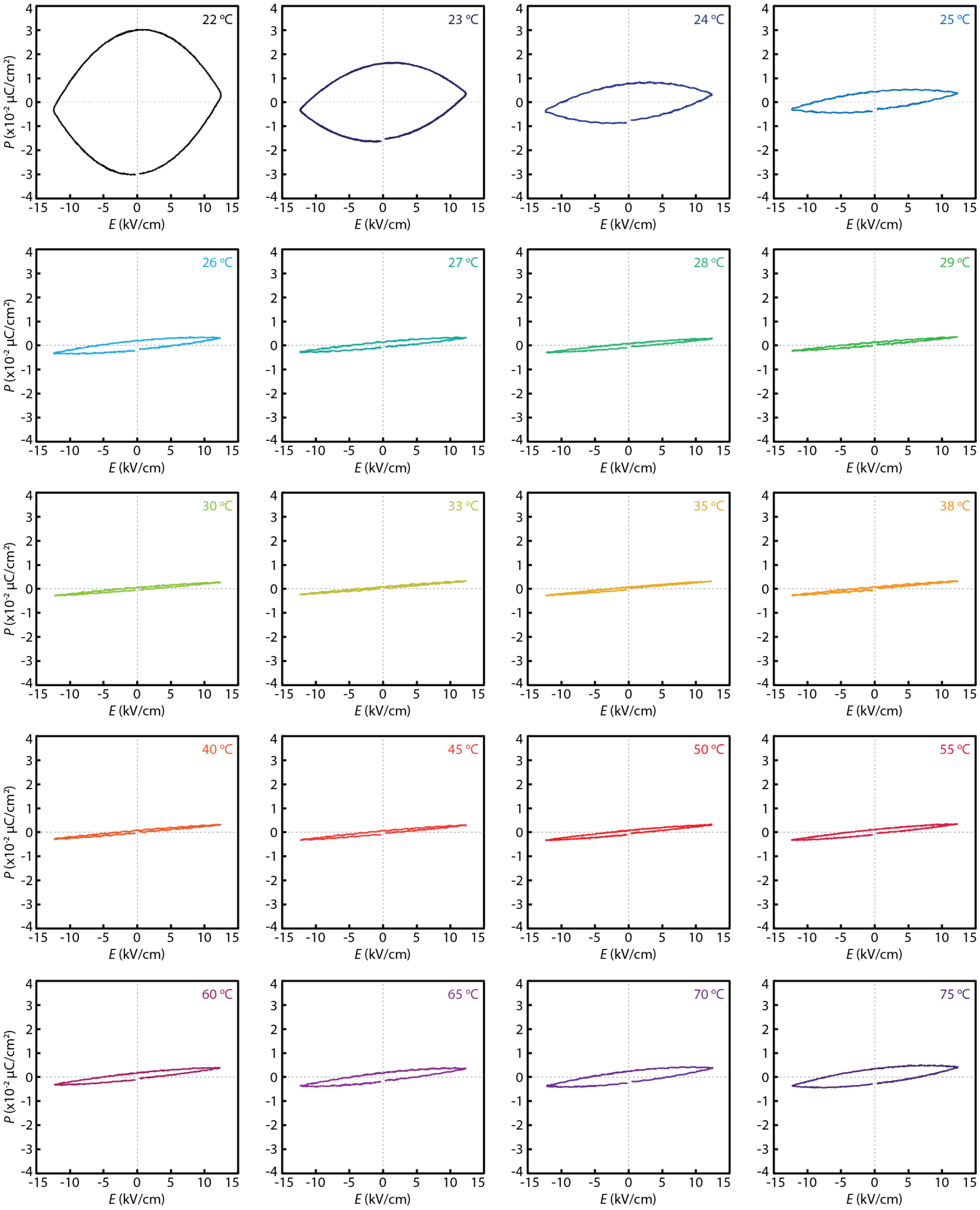

Figure SI 19. Polarization hysteresis loops for a single-crystalline device of desolvated DMDBS-DDQ (Device 1) with silver contacts placed on the (001) faces of the crystal. All loops were collected at 2 Hz at the temperatures indicated in the top right corner of each plot.

12.2. Temperature-Dependent Capacitive and Resistive Components for Device 1

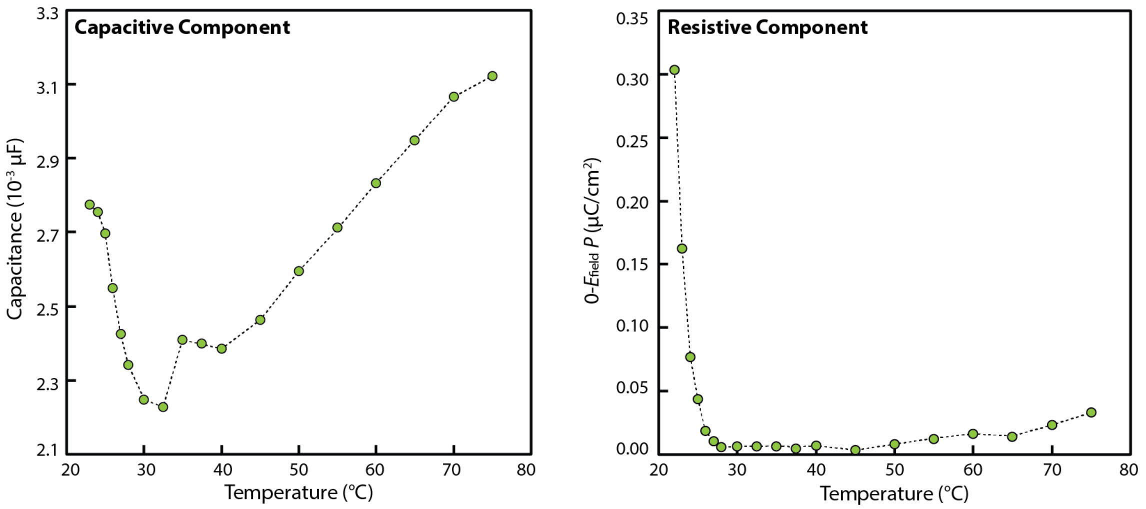

Figure SI 20. The capacitive and resistive components of the polarization hysteresis loops collected on Device 1 (Figure SI 19) plotted as a function of temperature. The capacitive component was extracted from the slope of the hysteresis loops and the resistive component was determined from the polarization magnitude measured at 0 kV/cm E_{field}.

12.3. Variable-Temperature Polarization Hysteresis Loops for Device 2

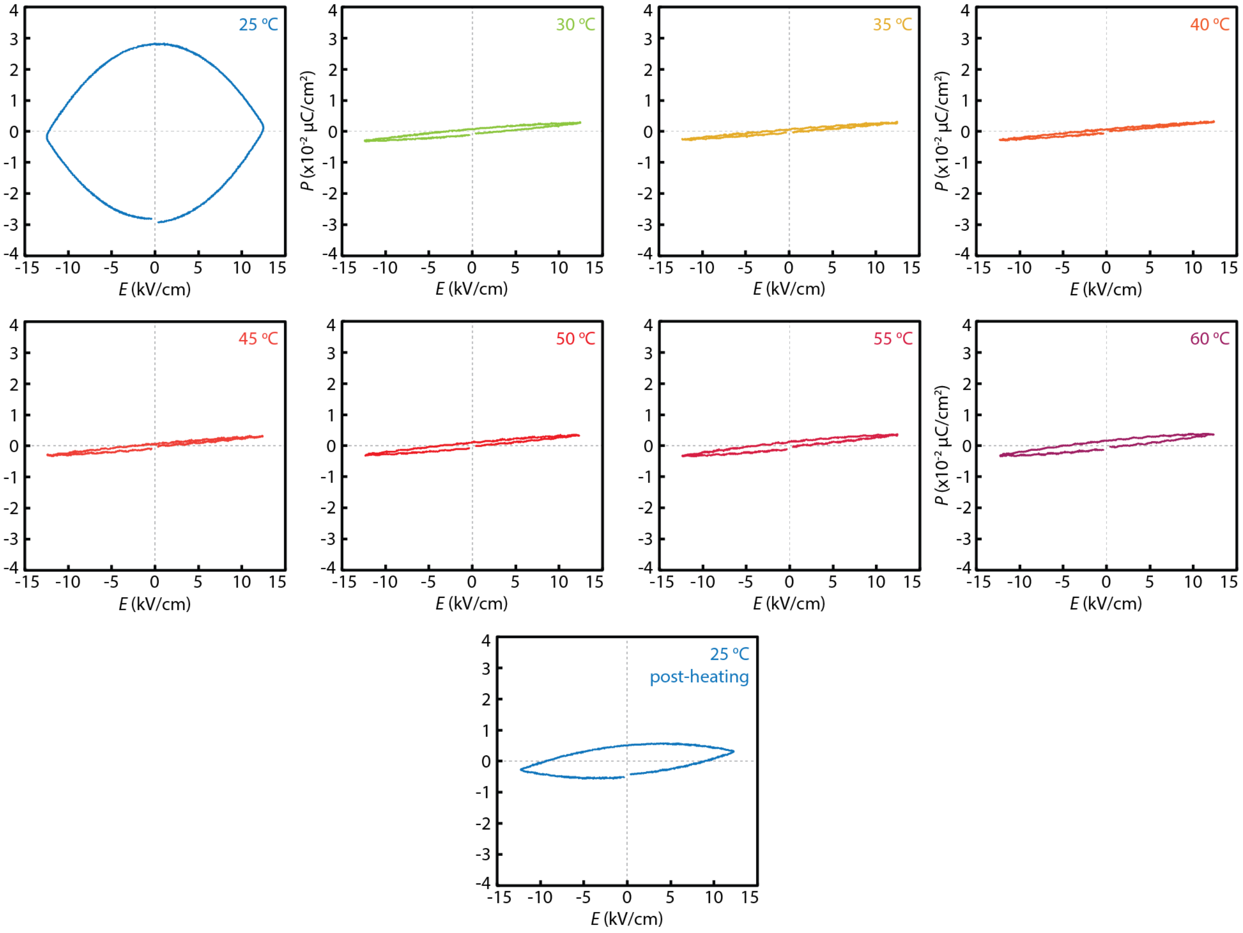

Figure SI 21. Polarization hysteresis loops for a second single-crystalline device of desolvated DMDBS-DDQ (Device 2) to investigate the reproducibility of the electrical behavior across multiple devices. Note that the initial 25 °C polarization hysteresis loop for Device 2 differs from Device 1; however, the behaviors of the two devices converge after thermal cycling to 70 °C. The difference is likely due to differences in defect densities following desolvation and the differences in device performance are reduced after thermal annealing. Silver contacts were placed on the (001) faces of the crystal. All loops were collected at 2 Hz at the temperatures indicated in the top right corner of each plot.

12.4. Temperature-Dependent Capacitive and Resistive Components for Device 2

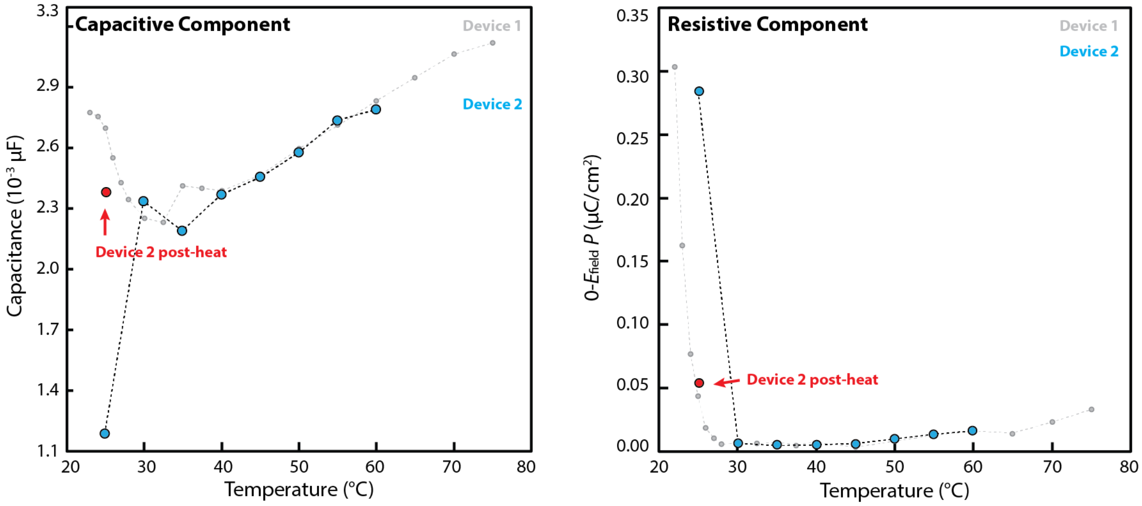

Figure SI 22. The capacitive and resistive components of the polarization hysteresis loops collected on Device 2 (Figure SI 21) plotted as a function of temperature. The capacitive component was extracted from the slope of the hysteresis loops and the resistive component was determined from the polarization magnitude measured at 0 kV/cm E_{field}. Note that the capacitive and resistive components extracted from the initial 25 °C polarization hysteresis loop for Device 2 differ from Device 1; however, the behaviors of the two devices converge after thermal cycling to 70 °C. The difference is likely due to differences in defect densities following desolvation and the differences in device performance are reduced after thermal annealing.

12.5. Cyclability of Polarization Loop Collapse for Device 1

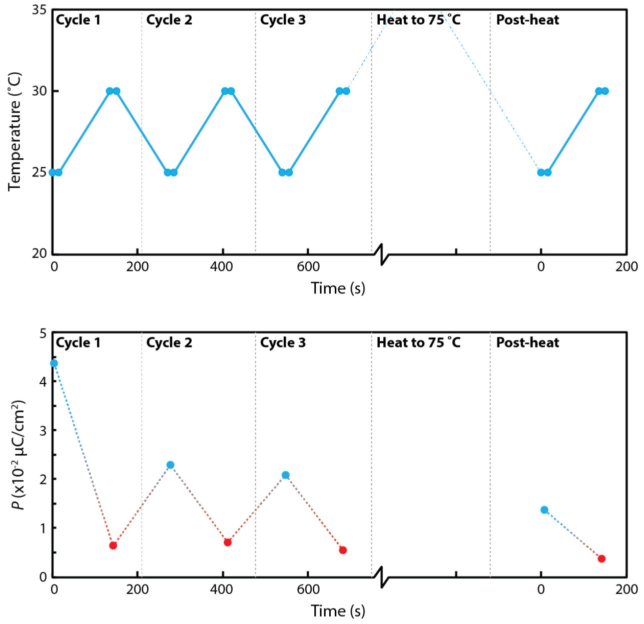

Figure SI 23. (Top) Temperature profile for the thermal cycling experiment between 25 and 30 °C. The device temperature was ramped at 2.5 °C/min and held stable over 15s for the polarization hysteresis measurement. The sample temperature was oscillated between 25 and 30 °C for three cycles before the sample temperature was ramped to 75 °C at 5 °C/min. Following heating of the sample to 75 °C, polarization hysteresis loops were again collected for the device at 25 and 30 °C ("Post-heat"). **(Bottom)** Polarization magnitude measured at 0-E_{field} for Device 1 for the three temperature oscillations between 25 and 30 °C and following heating of the sample to 75 °C, showing reproducibility of the polarization hysteresis loop collapse.

SI 13. Temperature-Dependent Lattice Constants for DMDBS-DDQ

Temperature	*a*-axis (Å)	*b*-axis (Å)	*c*-axis (Å)	α angle (°)	β angle (°)	γ angle (°)	Volume (Å^3)
**Crystal 1**							
100	6.4910	9.6103	16.490	76.890	88.982	73.147	957.5
125	6.4975	9.6213	16.526	76.928	88.92	73.036	961.3
150	6.5225	9.621	16.499	76.96	89.09	73.111	963.8
175	6.5284	9.6378	16.511	76.918	88.969	72.935	966.0
190	6.5377	9.6474	16.505	76.997	88.978	72.894	968.1
200	6.54195	9.64165	16.541	76.96	88.925	72.8475	969.95
210	6.5574	9.6509	16.507	77.102	88.916	72.832	971.7
225	6.5619	9.6561	16.568	77.006	88.907	72.773	975.7
250	6.5837	9.678	16.548	76.896	88.902	72.696	979.2
275	6.6049	9.6792	16.61	77.041	88.834	72.546	985.9
300	6.6267	9.6982	16.617	76.969	88.796	72.432	990.6
310	6.6434	9.7075	16.5915	76.967	88.844	72.501	992.89
320	6.6546	9.7094	16.627	77.049	88.666	72.373	996.7
330	6.6771	9.72	16.62	77.202	88.725	72.397	1001.5
340	6.686	9.756	16.601	77.24	88.9	72.01	1003.1
350	6.67	9.794	16.646	77.2	88.52	72.23	1008.7
**Crystal 2**							
325	6.6449	9.7057	16.6486	76.799	88.841	72.566	996.0
335	6.6579	9.7172	16.641	77.01	88.79	72.435	998.8
340	6.6778	9.738	16.649	76.91	88.77	72.43	1004.1
345	6.6682	9.759	16.637	77.4182	88.9644	72.2033	1004.7

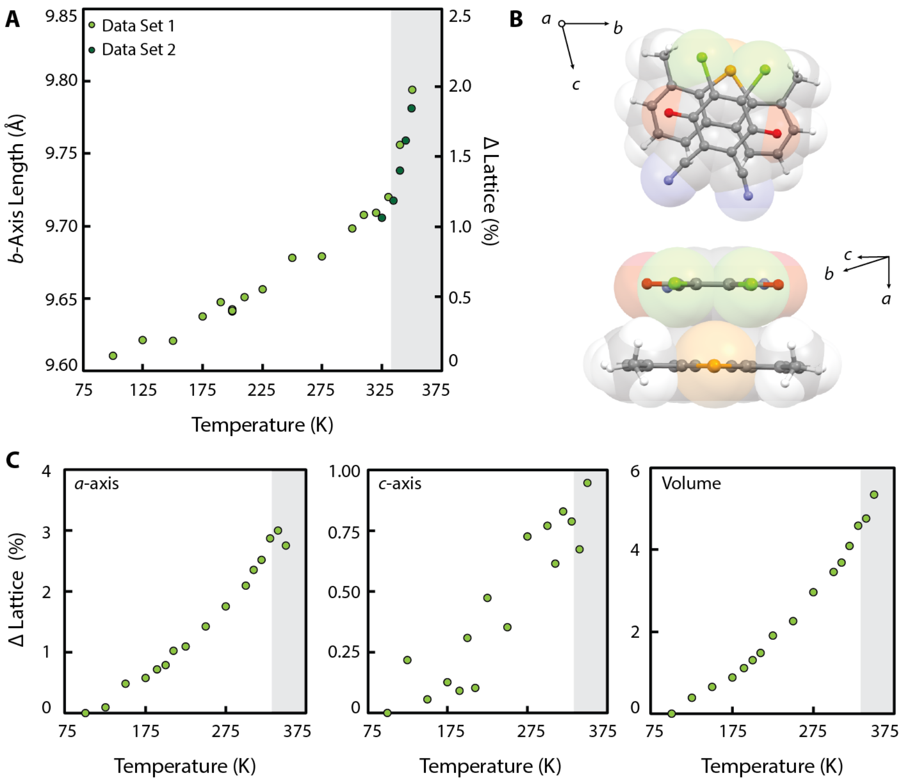

Figure SI 24. A) Plot of the DMDBS-DDQ *b*-axis length and change in the *b*-axis length relative to the lattice parameter measured at 100 K as a function of temperature. Data for two data sets are shown in green and blue; **B)** geometry of a DMDBS-DDQ π-stacked dimer relative to the unit cell axis directions; **C)** plots of the DMDBS-DDQ *a*-axis (left) and *c*-axis (middle) length changes and the unit cell volume relative to the parameter measured at 100 K as a function of temperature.

SI 14. Oak Ridge Thermal Ellipsoid Plots (ORTEP) for SCXRD Structures

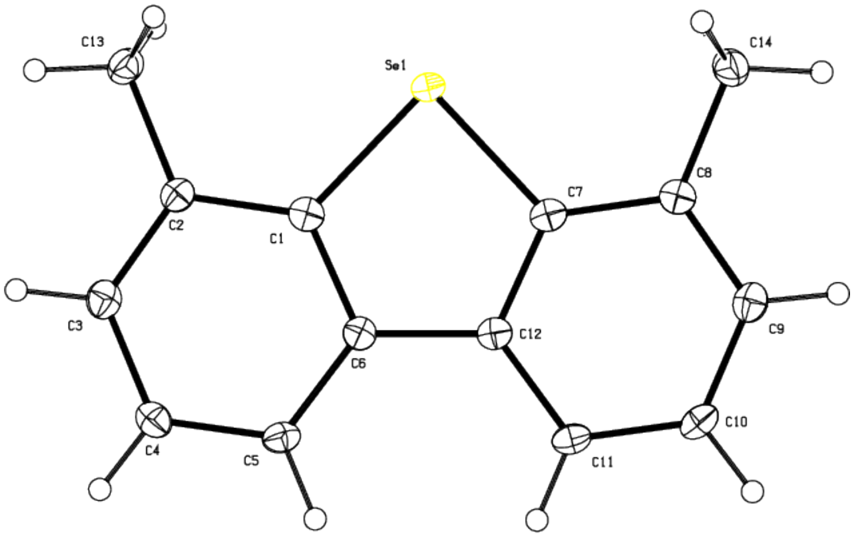

Figure SI 25. A view of the structure of DMDBS Form II showing the atom-labeling scheme. Displacement ellipsoids are drawn at the 30% probability level.

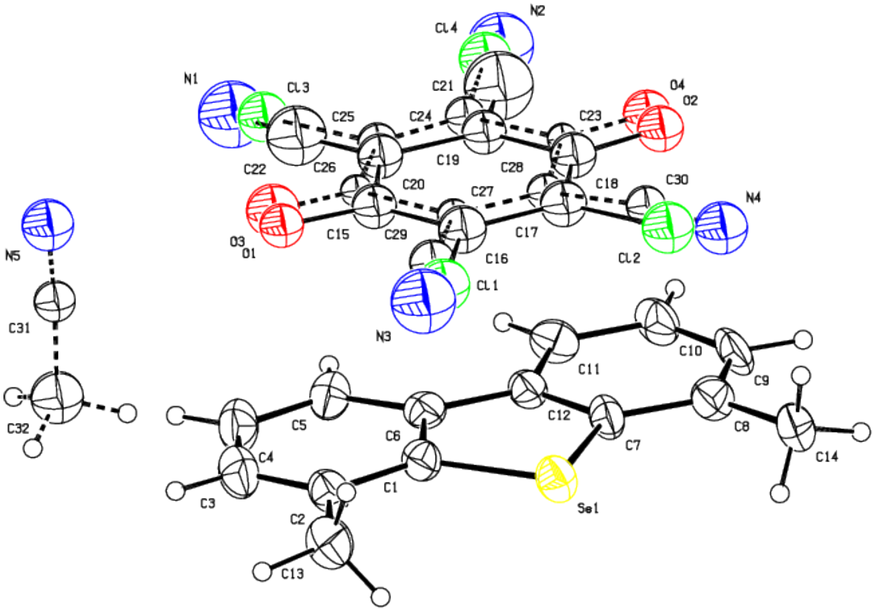

Figure SI 26. A view of the structure of DMDBS-DDQ-ACN$_X$ showing the atom-labeling scheme. Displacement ellipsoids are drawn at the 30% probability level.

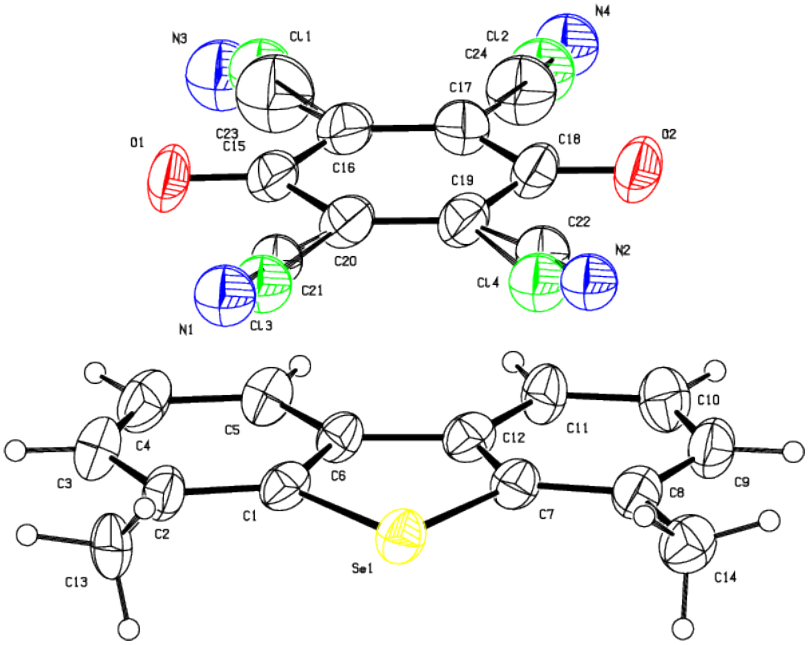

Figure SI 27. A view of the structure of DMDBS-DDQ showing the atom-labeling scheme. Displacement ellipsoids are drawn at the 30% probability level.

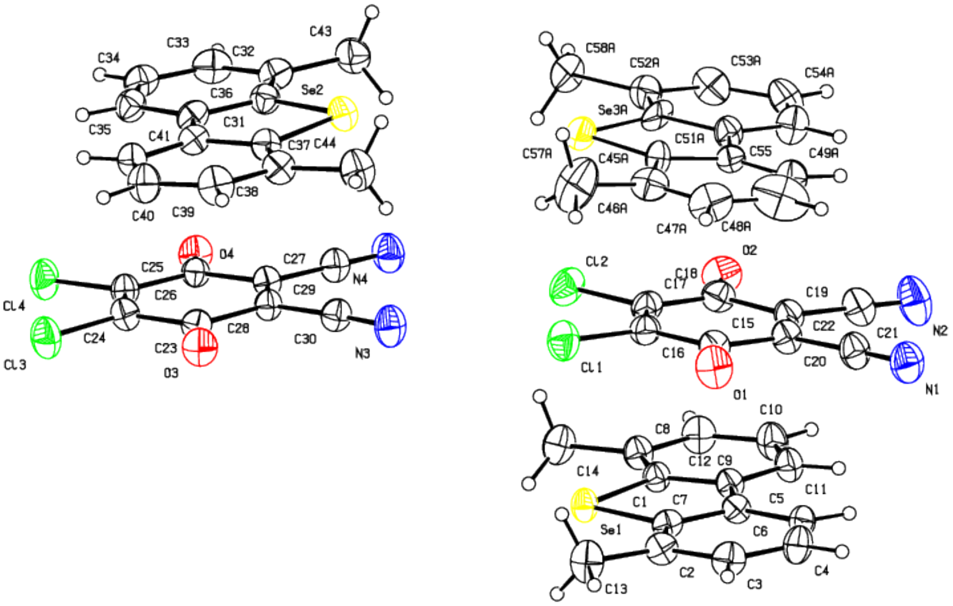

Figure SI 28. A view of the structure of $DMDBS_3$-DDQ_2, showing the atom-labeling scheme. Displacement ellipsoids are drawn at the 30% probability level.

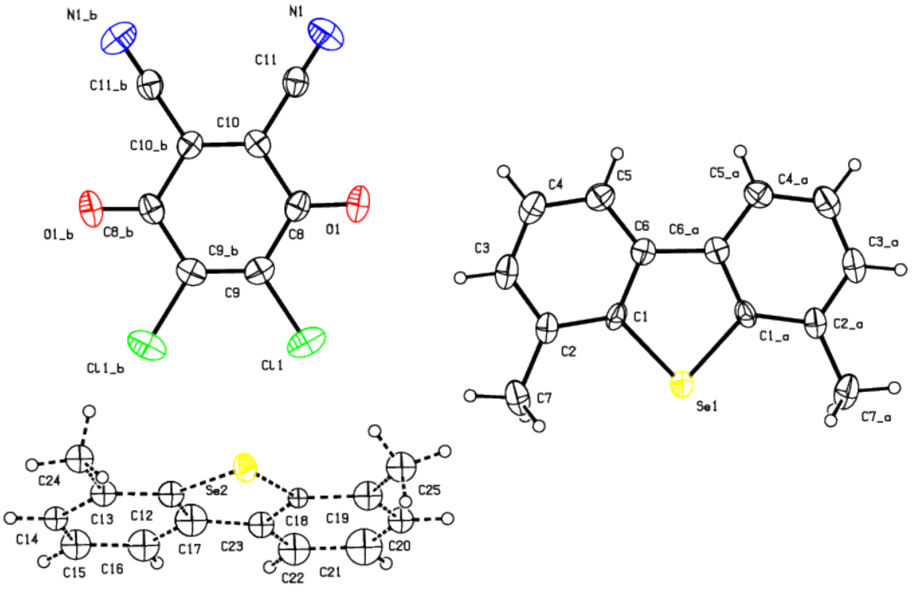

Figure SI 29. A view of the structure of DMDBS$_5$-DDQ$_4$, showing the atom-labeling scheme. Displacement ellipsoids are drawn at the 30% probability level.

SI 15. References

(1) Wiscons, R. A.; Coropceanu, V.; Matzger, A. J. Quaternary Charge-Transfer Solid Solutions: Electronic Tunability through Stoichiometry. *Chem. Mater.* **2019**, *31* (17), 6598–6604. https://doi.org/10.1021/acs.chemmater.9b00502.

(2) Dolomanov, O. V.; Bourhis, L. J.; Gildea, R. J.; Howard, J. a. K.; Puschmann, H. OLEX2: A Complete Structure Solution, Refinement and Analysis Program. *J. Appl. Crystallogr.* **2009**, *42* (2), 339–341. https://doi.org/10.1107/S0021889808042726.

(3) Sheldrick, G. M. *SHELXT* – Integrated Space-Group and Crystal-Structure Determination. *Acta Crystallogr. Sect. Found. Adv.* **2015**, *71* (1), 3–8. https://doi.org/10.1107/S2053273314026370.

(4) Sheldrick, G. M. Crystal Structure Refinement with SHELXL. *Acta Crystallogr. Sect. C Struct. Chem.* **2015**, *71* (1), 3–8. https://doi.org/10.1107/S2053229614024218.

(5) Macrae, C. F.; Bruno, I. J.; Chisholm, J. A.; Edgington, P. R.; McCabe, P.; Pidcock, E.; Rodriguez-Monge, L.; Taylor, R.; Streek, J. van de; Wood, P. A. Mercury CSD 2.0 – New Features for the Visualization and Investigation of Crystal Structures. *J. Appl. Crystallogr.* **2008**, *41* (2), 466–470. https://doi.org/10.1107/S0021889807067908.
